# The effect of an online video intervention ‘Movie Models’ on specific parenting practices and parental self-efficacy related to children’s physical activity, screen-time and healthy diet: a quasi experimental study

**DOI:** 10.1186/s12889-017-4264-1

**Published:** 2017-04-27

**Authors:** Sara De Lepeleere, Ilse De Bourdeaudhuij, Greet Cardon, Maïté Verloigne

**Affiliations:** Department of Movement and Sport Sciences, Ghent University, Watersportlaan 2, 9000 Ghent, Belgium

**Keywords:** Parenting, Parenting practices, Parental self-efficacy, Parent, Child, Physical activity, Screen-time, Healthy diet

## Abstract

**Background:**

In children, being sufficiently physically active, having low levels of screen-time and having a healthy diet are largely influenced by parenting practices. Children of parents applying positive parenting practices are at lower risk for overweight and obesity. Therefore, we investigated the effect of a health promoting online video intervention for parents (‘Movie Models’) on children’s physical activity (PA), screen-time and healthy diet, and on specific parenting practices and parental self-efficacy related to these parenting practices. The online videos are delivered to parents of primary schoolchildren, and were based on real-life scenarios.

**Methods:**

A two-armed, quasi experimental design was used. Parents of primary schoolchildren were recruited between November and December 2013 by spreading an appeal in social media, and by contacting primary schools. Participating parents were predominantly of high socio-economic status (SES) (83.1%), and only 6.8% of children were overweight/obese. Intervention group participants were invited to watch online videos for 4 weeks. Specific parenting practices, parental self-efficacy, PA, screen-time and healthy diet of the child were assessed at baseline (T0), at one (T1) and at four (T2) months post baseline. Repeated Measures (Multivariate) ANOVAs were used to examine intervention effects. The potential moderating effect of age and gender of the child and parental SES was also examined.

**Results:**

Between T0 and T2, no significant intervention effects were found on children’s PA, screen-time or healthy diet. Most significant intervention effects were found for more complex parenting practices (e.g., an increase in motivating the child to eat fruit). Subgroup analyses showed that the intervention had more effect on the actual parenting practices related to PA, screen-time and healthy diet in parents of older children (10–12 years old), whereas intervention effects on parental self-efficacy related to those behaviors were stronger in parents of younger children (6–9 years old).

**Conclusions:**

‘Movie Models’ was effective in increasing some important parenting practices and parental self-efficacy related to PA, screen-time and healthy diet in children. Therefore, the current study is an important first step in promoting effective parenting-related factors, and possibly increasing children’s healthy diet and PA, and decreasing screen-time.

**Trial registration:**

NCT02278809 in ClinicalTrials.gov on October 28, 2014 (retrospectively registered).

**Electronic supplementary material:**

The online version of this article (doi:10.1186/s12889-017-4264-1) contains supplementary material, which is available to authorized users.

## Background

Childhood overweight and obesity are associated with an avoidable burden of disease [1] such as cardiovascular diseases, diabetes, overweight/obesity, cancer, depression, fear, stress and poor self-image [2, 3] in adulthood. Being sufficiently physically active, having low levels of ‘screen-time’ (e.g., watching TV, playing video games), and having a healthy diet (i.e., eating a variety of foods which is essential to achieve adequate macro- and micronutrient intakes [4]), positively affect both physical and mental health, and can prevent overweight and obesity among children [5–7]. However, 83.2% of European boys and 95.4% of girls do not achieve the guidelines of at least 60 min of moderate to vigorous physical activity (PA) on most days of the week [8]. Furthermore, European children spend on average more than 2 h/day on screen time (TV and computer activities combined) [9], despite current guidelines recommending to spend no more than 2 h per day in recreational screen time [10]. European children also engage frequently in dietary behaviors that are regarded as potential risk behaviors for becoming overweight or obese. Many children skip breakfast on one or more days per week and the mean intake of sugar-sweetened beverages is high [9]. These data highlight the need for interventions designed to improve children’s healthy behaviors.

An increasing number of studies highlight the importance of parents in helping to shape healthy behaviors in their children [3, 11–14]. Parents may impact on their children’s behavior through *parenting practices* [15], which are specific practices of parents aiming to positively influence the child’s behavior, or through *parenting styles* [15], which refer to the broader emotional and relational climate in which these practices occur. Thus, parenting practices generally address what parents do, while parenting styles address how they do it. It is shown that parenting practices may be more effective when embedded in a positive parenting environment [16, 17]. Furthermore, literature shows that the authoritative parenting style, characterized by reasonable demands and high responsiveness, is associated with positive child and adolescent outcomes across multiple domains [11]. Therefore, parents can be an important focus of newly developed interventions to change children’s behavior.

Additionally, behavior change interventions that have been based on psychological theory tend to be more successful than those that have not [18]. Literature shows that Self Determination Theory (SDT) could provide a useful basis for interventions because of its conceptual similarity to the authoritative parenting style [11, 19]. According to SDT, all human beings have the fundamental need to feel related, competent and autonomous in order to develop and function optimally [20, 21]. Another important concept in SDT is internalization, the process by which individuals gradually transform certain externally reached beliefs, attitudes or behaviors into personally appreciated ones. As initially uninteresting activities become more internalized, they are performed with a larger feeling of autonomy, psychological freedom or self-determination [22]. However, evidence for effective, theory-based parenting interventions focusing on parenting practices to promote healthy behavior in children is still scarce [11, 23]. To date, most intervention programs have been situated within the school area because the existing organizational, social and communication structures provide opportunities for regular health education, and have the potential to reach children and their families across the social spectrum. Intervention programs try frequently to teach parents important parenting practices by distributing flyers, newsletters or homework tasks via their child’s school [24–26]. Unfortunately, a number of systematic reviews and meta-analyses have found that parents are difficult to reach with those strategies and interventions delivered within the school setting have shown limited effects [27–31]. This highlights a need for new, more effective approaches. In the study of Jago et al., parents of 6 to 8 year-old children attended an 8 week parenting program focusing on physical activity and screen-viewing (Teamplay). Each 2-h session consisted of three main topics and time for refreshments, games, parent feedback and the introduction of some tasks to be completed at home. Parenting aspects were aligned with SDT to encourage parents to use autonomy-supportive rather than controlling parenting strategies. Teamplay appeared to be a promising parenting program although it was highlighted that an internet-based maintenance program might be needed to maintain the intervention effects [19]. Furthermore, several benefits of using the internet in interventions are reported: being anonymous as a parent, information is available 24 h per day (most people have access to the internet 24/7 via their laptop, tablet, smartphone, etc.), the possibility to reach a wider audience and to increase access to organizations without an increase in costs [32]. Ruiter et al. also reported some strengths of a web-based e-learning program: parents can follow the program in their own home, at a time that suits best for them and at their own pace. Moreover, parents are not obliged to engage in a complex, time-consuming program [33]. Onnerfalt et al. developed a website for parents of preschool children to provide general information about nutrition and exercise recommendations, and also offered a parent support section. The website was used to prepare parents for a group intervention with the purpose of supporting them to accomplish preferred lifestyle changes, and to try new parenting techniques [34]. Also in the ‘Ehealth4Uth Healthy Toddler’ study of Raat et al., parents received an invitation to visit a website 1 month prior to the regular health care visit at child age 18 months. This way, parents obtained tailored parenting and health information on healthy child nutrition and activity behaviors. Furthermore, the general attitudes of the parents toward overweight prevention from early age on, and whether parents applied the recommended parental practices were examined. Results showed that the majority of the parents indeed understood the message, and viewed the message as useful and applicable [35]. Preliminary research in Flanders (Belgium) in which focus groups with parents and discussions with stakeholders and parenting experts were held, highlighted an interest in online videos as a way to learn how to perform effective parenting practices [36]. In light of the evidence reported above, an online intervention ‘Movie Models’ was developed. ‘Movie Models’ is based on the principles of SDT, and developed according to the Intervention Mapping Protocol [37]. The main goal of the intervention is to increase children’s PA, limit their screen-time, and improve their dietary behaviors, to prevent childhood overweight and obesity in the long run. Because parenting practices and parental self-efficacy might have a bridging function between the intervention and the child’s behavior, parenting practices and parental self-efficacy are examined as secondary outcomes.

Therefore, the first aim of the current study was to evaluate the effect of ‘Movie Models’ on the child’s behavior (PA, screen-time, healthy diet). Secondly, the intervention effect on parenting practices and parental self-efficacy was investigated. Finally, because it has been found that characteristics of parents and characteristics of the child that influence the parent-child relationship may influence parenting practices in the context of energy-related behaviors [11], we also examined if the intervention effects differed according to age and gender of the child and parental socio-economic status (SES).

## Methods

### Study design and setting

A two-armed, quasi experimental design was conducted in Flanders (i.e., the Dutch speaking part of Belgium). Ethical approval was provided by the Ethics Committee of the Ghent University Hospital (B670201214212).

### Participants and recruitment

A convenience sample of parents of primary schoolchildren was recruited by using different channels. Firstly, principals of 36 primary schools in Flanders were contacted personally by the researchers. In total, 30 schools (83%) agreed to participate. The only reason to decline was ‘not enough time’ (*n* = 6). In November–December 2013, flyers (*n* = 5077) to invite parents to participate were distributed in the participating schools to all 6- to 12-year old children to take home. However, because recruitment via schools was difficult, it was decided to also spread an appeal to participate by (social) media: two Flemish magazines for parents (‘Klasse’ and ‘De Gezinsbond’) and the Facebook page of EXPOO (expertise center for parenting support). Because it is unknown how many parents were reached by the invitation appeals, it is not possible to calculate a reliable response rate. Parents who wanted to participate had to send an email to the researcher. Afterwards, they were sent an information letter which contained information on the goal, the inclusion/exclusion criteria (having at least one primary schoolchild, availability of internet access at home, the primary school child is not on a diet nor has a physical disability), the content, the course, the starting and ending procedure, the risks, the advantages and confidentiality of the study. Furthermore, participants were sent the link to the online questionnaire, and were informed that by completing this online questionnaire, they gave consent to participate in the study. The recruitment of parents was ended by the second week of January 2014.

#### Development of the ‘Movie Models’ intervention

The development of the ‘Movie Models’ intervention, based on the Intervention Mapping Protocol, is described in detail elsewhere [38], but will be briefly discussed in this paper. After consultation of parents in focus group discussions and a parenting expert panel, 22 online videos about difficult parenting situations were developed. These videos (5 on PA, five on screen-time and 12 on healthy diet (water, breakfast, fruit, vegetables and buying healthy food in the supermarket)) each lasted about 2 min. In the videos, a difficult child-parent situation (concerning PA, screen-time or diet) is followed by an appropriate reaction of the parent (based on Self Determination Theory and Social Cognitive Theory which both support the principles of authoritative parenting). Afterwards, a narrator explains the parenting practices used in the video. This way, parents who watch the videos can learn effective parenting strategies related to PA, screen-time and diet usable in daily life through the modeling technique. Furthermore, parental attitude and parental self-efficacy concerning adopting the parenting strategies can be enhanced. The online videos are in Dutch, and can be watched on: www.gezondopvoeden.ugent.be. Table 1 shows a brief overview of the main messages of the 22 videos.

**Table 1:** Overview of the content of the 22 online parenting videos

Behavior	Video	Message	Week in which video was offered to parents
Water	Guideline	Video on the guideline for children to drink at least five big or six small glasses a day, why drinking water is important, …	1
	Tip 1	Let your child drink water limitless, and make rules concerning soft drink consumption (e.g., always drink water at dinner, except in weekends)	1
	Tip 2	Motivate your child to drink water (e.g., fill your child’s favorite goblet with water, and give it to him/her when he/she plays outdoors)	1
Fruit	Guideline	Video on the guideline for children to eat at least two or three pieces of fruit a day, why eating fruit is important, …	1
	Tip 1	Reinforce your child when he/she eats fruit (e.g., by giving a compliment)	1
	Tip 2	Make sure that healthy fruit snacks can be eaten easily (e.g., peel and cut the fruit into slice before giving it as a snack to school)	1
Vegetables	Guideline	Video on the guideline for children to eat at least five to twelve spoons of vegetables a day, why eating vegetables is important, …	2
	Tip 1	Motivate your child each time to taste a vegetable he/she does not like (e.g., it can be necessary to present a vegetable more than 10 times to your child before he/she likes it).	2
	Tip 2	Make sure that healthy vegetable snacks can be eaten easily (e.g., peel and cut the vegetable into slice before giving it as a snack to school)	2
Breakfast	Tip 1	Make sure breakfast is a peaceful and calm moment (e.g., the parent can make lunch the evening before so he/she can have breakfast together with the child.)	2
Supermarket	Tip 1	Involve your child in buying fruit and vegetables in the supermarket.	2
	Tip 2	Limit buying unhealthy food as much as possible (e.g., enter into agreements concerning buying cookies before going to the supermarket).	2
PA	Guideline	Video on the guideline for children to be physically active for at least 1 h a day, why PA is important, …	3
	Tip 1	Try to be active in as many ways as possible during the day (e.g., by accompanying your child by bike to school)	3
	Tip 2	Be a good model for your child by being physically active together with him/her (e.g., propose some activities you can do together).	3
	Tip 3	Motivate your child to be physically active (e.g., when he/she suddenly does not want to go to the youth movement anymore).	3
	Tip 4	Make physical activities pleasant for your child (e.g., let him/her choose to walk, roller-skate, ride a scooter…)	3
Screen-time	Guideline	Video on the guideline for children to limit screen time to 2 h a day, why sedentary behavior is unhealthy, …	4
	Tip 1	Enter into agreements about screen-time, and be consistent (e.g., let your child play on his/her Nintendo for 30 min, and make sure he/she stops playing after half an hour).	4
	Tip 2	Do not use TV or computer as a mean to keep your child calm (e.g., when you want to do the housekeeping).	4
	Tip 3	Do pleasant activities together with your child instead of watching TV (e.g., on a sunny Sunday afternoon you can frisbee, cycle, go to the park,…)	4
General	Time-out	Video on using time-out when your child really misbehaves.	4

#### Description of the ‘Movie Models’ intervention

After completing the online baseline questionnaire, participants were allocated to the intervention or control group (waitlist). Parents recruited via the appeal on social media were allocated randomly to the intervention or control group by computer randomization. Parents recruited within the same school were allocated to the same group (intervention or control) to avoid diffusion of intervention effects. Participants assigned to the intervention group were invited to watch the online parenting videos on a secured website over 4 weeks. At the start of the intervention period, the parenting videos concerning drinking water (*n* = 3) and eating fruit (*n* = 3) were put online. In the second week of the intervention, the videos concerning eating vegetables (*n* = 3), having breakfast (*n* = 1) and the supermarket (*n* = 2) were added to the website. During week three, parents could also watch the videos on PA (*n* = 5), and in week four the videos on screen-time (*n* = 5) were available. Intervention group parents were sent an invitation email every week to watch the new online videos. Furthermore, they were asked to fill out a short online process evaluation questionnaire (5–10 questions) on the videos that they watched the preceding week.

One (T1) and four (T2) months post baseline, both intervention and control group participants received the link to the same online questionnaire. The waiting list control group received no additional input during the period of the intervention, but got access to the online videos at the end of the study. As incentive, five gift vouchers were randomly distributed among parents who completed all questionnaires.

### Measures

At the beginning of the online questionnaire, it was mentioned that if parents had more than one child in primary school, they could choose for which child they wanted to complete the questionnaire. The questionnaire assessed demographic variables, parent-reported child’s PA, screen-time and healthy diet (primary outcome), specific parenting practices (secondary outcome) and parental self-efficacy concerning these practices (secondary outcome). All measures were assessed at baseline (T0), at the end of the intervention (T1, 1-month post baseline) and 3 months after the intervention had ended (T2, 4-months post baseline) to determine any change in parenting practices, parental self-efficacy and the behavior of the child.

#### Demographic variables

Age, gender, weight and height of the child, weight and height of both parents and number of children living in the house were reported in the questionnaire. The reported educational level of the parent who completed the questionnaire was used as a proxy for Socio-Economic Status (SES). Low SES was determined as parents having no higher education and medium to high SES as parents having higher education (vocational college, university or post-academic) [39]. Children’s body mass index (weight/height squared) was calculated from the parent-reported height and weight of the child. To define if a child was overweight or obese, age and sex specific cut off points developed by Cole et al. [40] for children from 2 to 18 years were used [40].

#### Child’s behavior

Levels of children’s PA and screen-time were assessed by the questionnaire adopted from the Flemish Physical Activity Questionnaire which has moderate criterion validity (Pearson correlation coefficient between 0.22 and 0.45 with accelerometers) [41, 42]. Total PA was assessed by adding up minutes spent in active transportation (to school and in leisure time) and time spent in sports (at school and during leisure time) per week. Screen-time was defined as the total time spent watching TV, playing computer games and using game consoles per week. The dietary behavior of the child was assessed by the Food Frequency Questionnaire which has a good criterion validity (Pearson correlation coefficients between 0.5 and 0.7 with estimated diet records) [43]. Consumption of fruit, vegetables, water, soft drinks and snacks were measured on a seven-point scale ranging from ‘1 = never’ to ‘7 = more than once every day’.

#### Specific parenting practices

Because we aimed to measure very specific parenting practices related to PA, screen-time and healthy diet, a new scale was developed based on the validated Parental Support For Physical Activity Scale (Cronbach’s alpha =0.78; test–retest reliability: *R* = 0.81) [44], the Parenting Strategies for Eating and Activity Scale [45] (Cronbach’s alpha = 0.81–0.82) and the Parental Feeding Style Questionnaire (Cronbach’s alpha = 0.67–0.83; test–retest reliability: *R* = 0.76–0.83) [46]. Most items were assessed on a two-point scale (disagree-agree) or a five-point Likert scale ranging from ‘never’ to ‘always’ (Additional file 1: Table S1).

#### Parental self-efficacy concerning the specific parenting practices

The parental self-efficacy questions were created analogous to the questions on the specific parenting practices, and were based on the translation of the GEMS (Girls Health Enrichment Multisite Study) questionnaire (Cronbach’s alpha = 0.52–0.62; test–retest reliability: *R* = 0.61–0.82) [47], the questionnaire of parental self-efficacy for enhancing healthy lifestyles in their children (Cronbach’s alpha = 0.94; test–retest reliability: *R* = 0.94) [48] and Section L of the Aventuras Para Ninos parent survey (Cronbach’s alpha = 0.73–0.87) [49]. The items were assessed by using a five-point answering format ranging from ‘completely disagree’ to ‘completely agree’ (Additional file 1: Table S1). These items were recoded to obtain a higher score when parents had a higher self-efficacy. The descriptive statistics of the specific parenting-related factors at baseline can be found elsewhere [50] (De Lepeleere S, Verloigne M, Cardon G, De Bourdeaudhuij I: Do Specific Parenting Practices and Parental Self-Efficacy associate with Beverages Intake among Belgian Primary Schoolchildren?, unpublished).

### Data analysis

Power analyses (powered on children’s PA, sedentary behavior and healthy diet, i.e., the primary outcomes of the study) revealed that 254 families were sufficient to investigate the possible effect of the ‘Movie Models’ intervention (power = 0.80; α = 0.01; effect size = 0.40).

Preliminary analyses consisted of descriptive statistics of sample characteristics and normality of key variables was checked. Because PA and screen-time at all three measurement moments were skewed, square root transformations were used to improve normality. For ease of interpretation, non-transformed mean values are reported in the tables.

Participants’ characteristics at baseline were compared by independent sample t-tests for quantitative variables and by chi-square tests for qualitative variables to detect baseline differences between the control and the intervention group, and to conduct a drop-out analysis. Because baseline characteristics did not differ significantly between intervention and control group, they were not used as covariates in further analyses. Intervention effects on children’s PA, screen-time, fruit, vegetable, water, soft drink and snack consumption were examined by conducting a Repeated Measures Multivariate ANOVA. Repeated Measures ANOVAs with time as within factor (differences between pre and posttest and between pre and follow-up) and condition (intervention group, control group) as between factor were conducted to examine intervention effects on parenting practices and parental self-efficacy. To examine potential moderating effects of children’s age (6–9 versus 10–12 years old), children’s sex (boys versus girls) and parental SES (low versus high SES), a three-way interaction effect (time*condition*moderator) was investigated for each outcome. In case of a significant three-way interaction effect for an outcome variable, analyses were stratified by the respective moderator. To consider the effect size of (borderline) significant interaction effects, we have reported Cohen’s d statistic (effect sizes 0.20–0.49 were considered small, 0.50–0.79 moderate and ≥0.80 large) [51]. Values are only reported in the text, not in the tables. All Repeated Measures (Multivariate) ANOVAs were performed using IBM SPSS Statistics 21.0. Due to the multiple tests that were performed, and to find a balance between Type I and Type II mistakes, only *p*-values <0.01 were considered significant. *P*-values ≥0.01 and <0.05 were considered borderline significant. All analyses were completer only analyses, and were conducted using SPSS (SPSS version 20.0, IBM corp., Armonk, NY; 2011). The dataset is available as Additional file 2: Table S4.

## Results

### Study characteristics

**Fig. 1:**
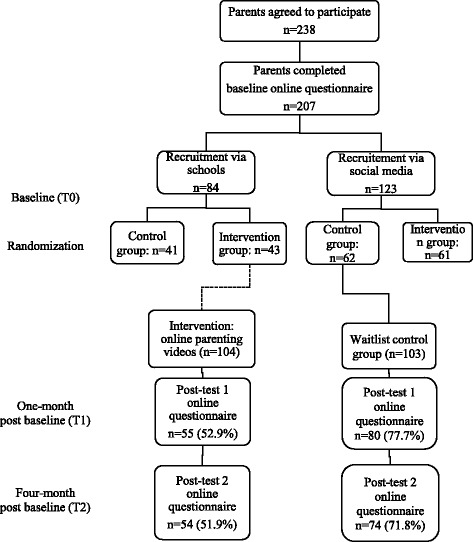
Participants flow through the study

The recruitment process resulted in 238 parents who agreed to participate. Of these parents, 207 (response rate of 87.0%) filled out the online questionnaire at baseline. Fifty-five (52.9%) intervention participants and 80 (77.7%) control participants completed 1-month post baseline measurements. Fifty-four (51.9%) intervention participants and 74 (71.8%) control participants completed 4-months post baseline measurements. Figure 1 shows the flow of participants through the study in detail. Based on data gathered in the online process evaluation questionnaire, each video was watched by 93.3–100% of parents from the intervention group. Consequently, it was not relevant to measure if intervention effects differed between parents who did and did not watch the videos.

Drop-out analyses indicated that participants from the intervention group (χ^2^ = 4.10, *p* = 0.04, two-tailed) were more likely to drop out. No significant differences were found for demographic variables, PA or screen-time of the child between parents who completed all measurements and parents who only filled out one or two questionnaires (Table 2).

**Table 2:** Drop-out analysis

Characteristic	Completers (*n* = 110)	Non Completers (*n* = 97)	Group comparison	*P*-value
Group, n (%)			χ^2^ = 4.10	**.04**
Intervention	48 (43.6)	56 (57.7)		
Control	62 (56.4)	41 (42.3)		
Demographic variable				
Gender parent, n (%)			χ^2^ = 0.02	.90
Male	13 (11.8)	12 (12.4)		
Female	97 (88.2)	85 (87.6)		
Age parent, mean (SD)	40.1 (4.3)	40.3 (5.7)	*t* = 0.28	.78
BMI mother, mean (SD)	23.8 (4.1)	23.8 (4.4)	*t* = −0.04	.97
BMI father, mean (SD)	25.0 (3.6)	24.6 (3.0)	*t* = −0.69	.49
SES, n (%)			χ^2^ = 0.05	.82
Low	18 (16.4)	17 (17.5)		
Medium-High	92 (83.6)	80 (82.5)		
Children’s PA level, mean minutes/day (SD)	54.1 (29.7)	48.5 (31.5)	*t* = −1.31	.19
Children’s screen-time, mean minutes/day (SD)	128.1 (73.4)	132.3 (78.3)	*t* = 0.39	.70

Significant *p*-values (*p*<0.05) are indicated in bold

Baseline characteristics of the intervention and control group are presented in Table 3. Control and intervention groups appear balanced on all demographic variables at baseline except for borderline significant differences in child’s age, number of children per family and baseline PA levels of the primary schoolchild (Table 3).

**Table 3:** Comparison of baseline characteristics

Characteristic	Intervention group (*n* = 104)	Control group (*n* = 103)	Group comparison	*P*-value
Demographic variable				
Gender parent, n (%)			χ^2^ = 0.06	.81
Male	12 (11.5)	13 (12.6)		
Female	92 (88.5)	90 (87.4)		
Gender child, n (%)			χ^2^ = 0.81	.37
Male	57 (54.8)	50 (48.5)		
Female	47 (45.2)	53 (51.5)		
Age parent, mean (SD)	40.5 (4.7)	39.9 (5.3)	*t* = −0.80	.43
Age child, mean (SD)	9.2 (1.5)	9.6 (1.6)	*t* = 1.94	***.05***
BMI mother, mean (SD)	23.5 (4.2)	24.1 (4.3)	*t* = 1.01	.31
BMI father, mean (SD)	24.9 (3.2)	24.7 (3.4)	*t* = −0.50	.62
SES, n (%)			χ^2^ = 0.05	.83
Low	17 (16.3)	18 (17.5)		
Medium-High	87 (83.7)	85 (82.5)		
Number of children per family, mean (SD)	2.2 (0.9)	2.4 (0.7)	*t* = 1.77	***.08***
Children’s PA level, mean minutes/day (SD)	46.6 (27.0)	55.3 (33.5)	*t* = 1.81	***.07***
Children’s screen-time, mean minutes/day (SD)	122.9 (66.0)	137.1 (83.7)	*t* = 1.32	.19

Significant p-values (*p*<0.05) are indicated in bold; borderline significant *p*-values (0.05 ≤ *p*<0.10) are indicated in bold italic

### Intervention effects on children’s behavior

The Repeated Measures MANOVA analyses demonstrated that the intervention had no effect on children’s health behaviors reported by parents between baseline and 1-month follow-up (F = 0.15; *p* = 0.99) and between baseline and 4-months follow-up (F = 0.79; *p* = 0.59) (Additional file 3: Table S2).

In the following paragraph, the intervention effects on parenting practices and parental self-efficacy are described. Only the significant and borderline significant results are reported in the text, and can be found in Table 4. The non-significant results can be found in Additional file 3: Table S2.

**Table 4:** Significant and borderline significant two-way and three-way interaction effects on parenting practices and parental self-efficacy

Parenting practices from T0 to T1												
Univariate		n	T0	T1	Time x Group		Time x Group x Age		Time x Group x Gender		Time x Group x Parental SES	
			Mean (SD)	Mean (SD)	F	P	F	P	F	P	F	P
Involving in household chores	IG	44	3.20 (0.77)	3.30 (0.77)	4.78	***0.031***	3.09	0.082	0.34	0.559	0.20	0.653
	CG	70	3.04 (0.84)	2.87 (0.90)								
Rules concerning TV	IG	41	0.78 (0.42)	0.85 (0.36)	0.00	0.971	2.02	0.159	0.49	0.486	6.48	***0.012***
	CG	66	0.80 (0.40)	0.88 (0.33)								
Rules concerning gaming	IG	37	0.78 (0.42)	0.86 (0.35)	1.16	0.285	2.88	0.093	0.00	0.968	4.62	***0.034***
	CG	61	0.75 (0.43)	0.92 (0.28)								
Modeling concerning TV	IG	45	4.00 (0.95)	3.91 (0.97)	1.79	0.183	3.24	0.075	1.18	0.280	5.50	***0.021***
	CG	69	3.43 (1.16)	3.59 (1.05)								
Modeling concerning gaming	IG	45	3.89 (0.93)	3.60 (0.96)	2.42	0.123	0.01	0.905	0.70	0.403	7.80	**0.006**
	CG	69	3.35 (0.98)	3.35 (0.94)								
Permissiveness concerning water (how much)	IG	50	4.32 (0.98)	4.58 (0.70)	3.56	0.062	0.09	0.764	1.44	0.233	4.32	***0.040***
	CG	69	4.62 (0.71)	4.48 (0.87)								
Being consistent concerning soft drinks	IG	31	4.48 (0.51)	4.45 (0.51)	0.05	0.826	4.43	***0.039***	1.57	0.215	0.56	0.456
	CG	42	4.48 (0.51)	4.40 (0.59)								
Parenting practices from T0 to T2												
Univariate		n	T0	T2	Time x Group		Time x Group x Age		Time x Group x Gender		Time x Group x Parental SES	
			Mean (SD)	Mean (SD)	F	P	F	P	F	P	F	P
Modeling of PA	IG	49	3.27 (1.13)	3.27 (1.09)	0.00	1.000	0.34	0.563	1.12	0.291	4.64	***0.034***
	CG	63	3.19 (1.19)	3.19 (1.13)								
Being consistent concerning TV	IG	37	4.03 (0.55)	1.19 (0.66)	0.73	0.396	6.75	***0.011***	1.06	0.306	0.72	0.399
	CG	45	3.89 (0.57)	3.93 (0.58)								
Giving an explanation concerning TV	IG	37	4.22 (0.85)	4.24 (0.83)	0.10	0.750	9.06	**0.004**	1.88	0.175	0.02	0.891
	CG	45	4.09 (0.85)	4.18 (0.68)								
Giving an explanation concerning gaming	IG	28	4.43 (0.69)	4.32 (0.82)	0.29	0.591	6.65	***0.012***	0.59	0.444	0.29	0.593
	CG	40	4.08 (0.86)	4.08 (0.73)								
Monitoring gaming	IG	40	3.40 (1.30)	3.85 (0.92)	1.84	0.178	2.63	0.108	2.40	0.125	5.10	***0.026***
	CG	54	3.57 (1.08)	3.72 (1.00)								
Modeling concerning TV	IG	50	3.96 (0.97)	4.16 (0.98)	1.57	0.213	0.31	0.578	0.01	0.906	5.14	***0.025***
	CG	63	3.49 (1.20)	3.46 (1.15)								
Motivating concerning fruit	IG	51	4.04 (1.26)	4.47 (1.03)	8.00	**0.006**	0.24	0.624	0.18	0.677	1.69	0.196
	CG	66	4.08 (1.13)	3.79 (1.21)								
Permissiveness concerning how much vegetables between meals	IG	50	3.70 (1.22)	3.94 (1.06)	1.67	0.198	4.54	***0.035***	0.41	0.524	0.01	0.930
	CG	66	4.02 (1.10)	4.00 (0.93)								
Permissiveness concerning water (when)	IG	50	4.70 (0.79)	4.58 (0.79)	0.10	0.749	6.86	***0.010***	0.01	0.914	3.57	0.061
	CG	66	4.79 (0.60)	4.71 (0.58)								
Choice concerning water	IG	52	2.87 (1.52)	2.83 (1.41)	0.56	0.455	0.02	0.885	0.13	0.723	6.08	***0.015***
	CG	66	3.24 (1.55)	3.36 (1.41)								
Being consistent concerning soft drinks	IG	34	4.38 (0.49)	4.41 (0.61)	0.00	0.950	0.09	0.761	1.75	0.189	8.43	**0.005**
	CG	47	4.45 (0.54)	4.47 (0.58)								
Giving an explanation concerning soft drinks	IG	34	4.59 (0.70)	4.29 (0.72)	0.17	0.677	4.08	***0.047***	0.60	0.440	0.88	0.351
	CG	46	4.59 (0.65)	4.22 (0.92)								
Parental self-efficacy from T0 –toT1												
Univariate		n	T0	T1	Time x Group		Time x Group x Age		Time x Group x Gender		Time x Group x Parental SES	
			Mean (SD)	Mean (SD)	F	P	F	P	F	P	F	P
SE Modeling of PA	IG	44	3.16 (1.38)	3.64 (1.30)	4.05	***0.046***	0.03	0.862	1.00	0.319	0.00	0.990
	CG	69	3.13 (1.40)	3.25 (1.29)								
SE Giving choice for PA	IG	44	4.39 (0.75)	4.25 (0.94)	1.07	0.304	4.52	***0.036***	0.28	0.598	0.03	0.875
	CG	67	4.33 (0.88)	4.36 (0.85)								
SE Modeling concerning gaming	IG	44	4.07 (1.00)	4.05 (1.01)	1.11	0.294	6.25	***0.014***	0.00	0.959	1.05	0.307
	CG	70	4.16 (0.96)	3.93 (0.98)								
SE Motivating concerning fruit	IG	23	3.48 (1.27)	3.57 (1.31)	0.35	0.557	3.88	0.054	1.45	0.234	4.85	***0.032***
	CG	35	3.09 (1.44)	3.46 (1.27)								
SE Choice concerning fruit	IG	46	4.20 (1.02)	4.43 (0.83)	1.12	0.292	10.06	**0.002**	0.11	0.738	0.98	0.325
	CG	67	4.46 (0.86)	4.46 (0.64)								
SE Availability of fruit	IG	47	4.70 (0.69)	4.79 (0.55)	0.79	0.375	5.64	***0.019***	0.00	0.979	3.85	0.052
	CG	67	4.84 (0.41)	4.81 (0.47)								
SE Modeling concerning vegetables	IG	46	4.87 (0.40)	4.87 (0.40)	0.87	0.353	0.28	0.599	5.69	***0.019***	0.20	0.658
	CG	68	4.88 (0.37)	4.79 (0.48)								
SE Availability of vegetables	IG	47	4.72 (0.54)	4.77 (0.56)	0.01	0.932	9.63	**0.002**	0.21	0.650	1.26	0.264
	CG	67	4.73 (0.59)	4.77 (0.56)								
SE Giving an explanation concerning soft drinks	IG	30	4.57 (0.63)	4.63 (0.56)	0.28	0.601	4.27	***0.043***	8.62	**0.005**	0.00	0.968
	CG	42	4.57 (0.74)	4.50 (0.92)								
Parental self-efficacy from T0 to T2												
Univariate		n	T0	T2	Time x Group		Time x Group x Age		Time x Group x Gender		Time x Group x Parental SES	
			Mean (SD)	Mean (SD)	F	P	F	P	F	P	F	P
SE Permission concerning gaming	IG	50	4.30 (0.87)	4.20 (1.09)	0.78	0.380	4.25	***0.042***	1.06	0.306	0.44	0.509
	CG	65	4.35 (0.87)	4.06 (1.03)								
SE Involving concerning fruit	IG	51	4.29 (0.88)	4.45 (1.03)	0.72	0.398	4.85	***0.030***	0.94	0.334	0.02	0.899
	CG	67	4.55 (0.82)	4.54 (0.78)								
SE Motivating concerning vegetables	IG	41	3.05 (1.38)	3.66 (1.22)	4.95	***0.029***	0.27	0.608	0.32	0.576	2.25	0.137
	CG	51	3.41 (1.37)	3.51 (1.25)								
SE Availability of vegetables	IG	51	4.78 (0.46)	4.69 (0.58)	4.79	***0.031***	0.08	0.785	0.05	0.823	0.02	0.903
	CG	66	4.68 (0.64)	4.79 (0.51)								
SE Involving concerning vegetables	IG	51	4.08 (1.07)	4.18 (1.18)	1.30	0.257	6.00	***0.016***	1.91	0.169	2.20	0.141
	CG	66	4.48 (0.85)	4.35 (0.95)								
SE Permissiveness concerning water (when)	IG	48	4.17 (1.33)	4.56 (0.71)	6.07	***0.015***	0.08	0.777	0.00	0.996	0.11	0.742
	CG	62	4.73 (0.58)	4.63 (0.79)								

*IG* intervention group, *CG* control group, *SE* self-efficacy

Significant *p*-values are indicated in bold; borderline significant *p*-values are indicated in bold italic

All intervention effects (significant and non-significant) can be found in Additional file 3: Table S2

### Intervention effects on parenting practices

Regarding PA and screen-time, the two-way interaction effects showed that the intervention-parents had a borderline significant increase for ‘involving your child in household chores’ (F = 4.78; *p* = 0.03; d = 0.33) at 1-month follow-up compared to the control group. No significant intervention effects were found on parenting practices concerning fruit, vegetable, water, soft drink and snack consumption between baseline and 1-month follow-up (Additional file 3: Table S2).

Between baseline and 4-months follow-up, there was only a significant positive intervention effect on ‘motivating your child to eat fruit’ (F = 8.00; *p* = 0.006; d = 0.61) (Additional file 3: Table S2).

### Intervention effects on parental self-efficacy related to parenting practices

Intervention parents had a borderline significant increase in their ‘self-efficacy for being physically active themselves’ (F = 4.05; *p* = 0.046; d = 0.26) at 1-month follow-up compared to the control group (Additional file 3: Table S2).

At 4-months follow-up, intervention group parents had a borderline significant increase in their ‘self-efficacy for motivating your child to eat vegetables’ (F = 4.95; *p* = 0.03; d = 0.37) and ‘self-efficacy for giving your child as much freedom as possible to drink water’ (F = 6.07; *p* = 0.02; d = 0.50) compared to control group parents. Unexpectedly, parents of the intervention group had a significant decrease in their ‘self-efficacy concerning having vegetables available’ (F = 4.79; *p* = 0.03; d = −0.35) after 4 months (Additional file 3: Table S2).

### Moderating effects of child’s age, gender and parental SES

#### Child’s age

##### Parenting practices

From baseline to 1-month follow-up, the time*group*age interaction effect was borderline significant for ‘following up your rules concerning soft drink consumption’ (F = 4.43; *p* = 0.04) (Additional file 3: Table S2). However, stratified analyses showed no significant intervention effects for younger or older children (Additional file 4: Table S3).

From baseline to 4-months follow-up, the time*group*age interaction effect was significant or borderline significant for ‘following up your rules concerning TV-time’ (F = 6.75; *p* = 0.011), ‘giving an explanation concerning TV’ (F = 9.06; *p* = 0.004), ‘giving an explanation concerning gaming’ (F = 6.65; *p* = 0.012), ‘permissiveness concerning how much vegetables your child is allowed to eat between meals’ (F = 4.54; *p* = 0.035), ‘permissiveness concerning when your child is allowed to drink water’ (F = 6.86; *p* = 0.010) and ‘giving an explanation concerning soft drinks’ (F = 4.08; *p* = 0.047) (see Additional file 3: Table S2). Stratified analyses showed that there were only significant or borderline significant intervention effects among parents of older children (10–12 year old): intervention group parents reported less ‘giving an explanation concerning TV’ (F = 5.37; *p* = 0.03; d = −0.85) and ‘giving an explanation concerning gaming’ (F = 5.04; *p* = 0.03; d = −0.81), and reported more ‘permissiveness concerning how much vegetables your child is allowed to eat between meals’ (F = 11.70; *p* < 0.001; d = 0.76) 4 months later (Additional file 4: Table S3).

##### Parental self-efficacy

From baseline to 1-month follow-up, the time*group*age interaction effect was significant or borderline significant for ‘parental self-efficacy concerning giving choice for PA’ (F = 4.52; *p* = 0.04), ‘limiting your own gaming (modeling)’ (F = 6.25; *p* = 0.014), ‘letting your child choose between different kinds of fruit’ (F = 10.06; *p* = 0.002), ‘availability of fruit’ (F = 5.64; *p* = 0.02), ‘availability of vegetables’ (F = 9.63; *p* = 0.002) and ‘giving an explanation concerning soft drinks’ (F = 4.27; *p* = 0.04) (Additional file 3: Table S2). Stratified analyses showed that there were only significant or borderline significant intervention effects among parents of younger children (6–9 year old): intervention group parents reported a higher ‘self-efficacy concerning limiting your own gaming’ (F = 6.47; *p* = 0.014; d = 0.62), ‘self-efficacy concerning letting your child choose between different kinds of fruit’ (F = 8.38; *p* = 0.005; d = 0.97), ‘self-efficacy concerning having fruit available’ (F = 5.06; *p* = 0.03; d = 0.66) and ‘self-efficacy concerning having vegetables available’ (F = 5.80; *p* = 0.02; d = 0.79) at 1 month follow-up (Additional file 4: Table S3).

From baseline to 4-months follow-up, the time*group*age interaction effect was borderline significant for ‘parental self-efficacy concerning letting your child ask for permission to play games’ (F = 4.25; *p* = 0.04), ‘self-efficacy concerning involving your child in buying fruit’ (F = 4.85; *p* = 0.03) and ‘self-efficacy concerning involving your child in buying vegetables’ (F = 6.00; *p* = 0.02). Stratified analyses showed that there were only borderline significant intervention effects among parents of younger children (6–9 year old): intervention group parents reported a higher self-efficacy concerning letting your child ask for permission to play games (F = 4.95; *p* = 0.03; d = 0.64) and concerning involving your child in buying vegetables (F = 5.56; *p* = 0.02; d = 0.73) after 4 months (Additional file 4: Table S3).

#### Child’s gender

The time*group*gender interaction effect was only significant or borderline significant for ‘parental self-efficacy on eating vegetables in front of your child (modeling)’ (F = 5.69; *p* = 0.02) and ‘parental self-efficacy on giving your child an explanation why there are rules about soft drinks’ (F = 8.62; *p* = 0.005) from baseline to 1-month follow-up (Additional file 3: Table S2). Stratified analyses showed that there was only a borderline significant intervention effect for ‘parental self-efficacy concerning modeling of vegetables’ among parents of boys (F = 6.38; *p* = 0.014; d = 0.79): intervention group parents had a higher self-efficacy after the intervention. In contrast, the intervention effect for ‘parental self-efficacy on giving your child an explanation why there are rules about soft drinks’ was only borderline significant among parents of girls (F = 4.44; *p* = 0.043; d = 1.33): intervention group parents had a higher self-efficacy after the intervention (Additional file 4: Table S3).

#### Parental SES

##### Parenting practices

From baseline to 1-month follow-up, the time*group*SES interaction effect was significant or borderline significant for ‘rules concerning TV-time’ (F = 6.48; *p* = 0.012), ‘rules concerning gaming’ (F = 4.62; *p* = 0.03), ‘limiting your own TV-time’ (F = 5.50; *p* = 0.02), ‘limiting your own gaming’ (F = 7.80; *p* = 0.006) and ‘letting your child choose how much water he/she wants to drink’ (F = 4.32; *p* = 0.04). Stratified analyses showed no significant intervention effects on applying rules for TV-time or gaming among low or high SES families. Stratified analyses showed that there were only borderline significant intervention effects for ‘limiting your own TV-time’ (F = 5.75; *p* = 0.03; d = −1.23) and ‘limiting your own gaming’ (F = 6.67; *p* = 0.02; d = −2.14) among low SES families: intervention group parents had a lower performance of the parenting practices after the intervention. For ‘letting your child choose how much water he/she wants to drink’ (F = 7.16; *p* = 0.009; d = 0.74) there was only a significant intervention effect among high SES families: intervention group parents were more permissive after the intervention (Additional file 4: Table S3).

From baseline to 4-months follow-up, the time*group*SES interaction effect was significant or borderline significant for ‘being physically active yourself (modeling)’ (F = 4.64; *p* = 0.03), ‘monitoring gaming’ (F = 5.10; *p* = 0.03), ‘limiting your own TV-time (modeling)’ (F = 5.14; *p* = 0.03), ‘being consistent concerning soft drinks’ (F = 8.43; *p* = 0.005) and ‘letting your child choose between different kinds of water’ (F = 6.08; *p* = 0.02) (Additional file 3: Table S2). Stratified analyses showed that there was only a borderline significant intervention effect for ‘letting your child choose between different kinds of water’ (F = 5.87; *p* = 0.03; d = −0.95) among low SES families: intervention group parents had a lower performance of the parenting practice after the intervention. On the other hand, there was only a borderline significant intervention effect for ‘monitoring gaming’ (F = 4.68; *p* = 0.03; d = 0.46) among high SES families: intervention group parents monitored their children more after the intervention. For ‘modeling of PA’, ‘modeling concerning TV-time’ and ‘being consistent concerning soft drinks’, no significant intervention effects for low or high SES families were found (Additional file 4: Table S3).

##### Self-efficacy

From baseline to 1-month follow-up, the time*group*SES interaction effect was statistically significant for ‘parental self-efficacy concerning motivating your child to eat fruit’ (F = 4.85; *p* = 0.03) (Additional file 3: Table S2). However, stratified analyses showed no significant intervention effects for low or high SES families (Additional file 4: Table S3).

## Discussion

The current study investigated the effect of the online video intervention ‘Movie Models’ on children’s PA, screen-time and healthy diet reported by parents; on specific parenting practices; and on parental self-efficacy related to these parenting practices. We also examined the potential moderating effects of child’s age and gender and parental SES on the intervention effect.

The analyses demonstrated that the ‘Movie Models’ intervention had no effect on children’s health behavior immediately and 3 months after the intervention. It could be that the follow-up period of measurement is too short. Because parental factors (parenting practices and parental self-efficacy) are a bridging function between the intervention and the child’s behavior, it is possible that a longer period of time is necessary to change the child’s health behavior. It is conceivable that if the changes in parenting practices and parental self-efficacy sustained over a longer time, changes in children’s behavior may still occur. Also in the study of Naylor et al. (2016), changing parenting cognitions and practices in the home showed no effects on children’s vegetable consumption, possibly also because of the short follow-up measurement period [52]. We would expect more effects on parenting practices and parental self-efficacy as the intervention specifically targeted those factors, but only a limited number of effects were found. Nevertheless, a clear tendency was found: the significant intervention effects were mainly on the more complex and less concrete parenting-related factors such as ‘how you can motivate your child to eat fruit’. The parenting-related factors on which the intervention had no effect seemed to be rather simple, more concrete and more obvious such as ‘having sports material at home’, ‘reinforcing your child to eat fruit or vegetables, to drink water, to be physically active,…’ and ‘explaining your child why drinking water is important’. Furthermore, although there was only a limited number of effects, most effects were of moderate size. Therefore, this can be considered a positive and important finding of the intervention.

Several explanations can be given for the larger number of effects that were found on parenting-related factors related to healthy diet compared to PA and screen-time in the total sample. Firstly, the videos on healthy diet were put online in the first and second week of the intervention, and parents were still highly motivated in that period to apply the specific parenting practices in real life. Also literature shows that the interest in a web based program is higher at the start of the program [53]. Next, parenting practices and parental self-efficacy related to healthy diet might be easier to integrate in one’s daily life because people eat at least three times a day, while PA and screen-time might be seen as supplemental to the biological needs such as feeding. Finally, also the review of Golley et al. showed that intervention effectiveness was in favor of interventions targeting energy intake/density and food choices [23].

Although the effects were rather limited in the total sample, more intervention effects were found in certain subgroups of parents. When considering the child’s age as a moderator, the intervention had effect on six parenting-related factors in younger children (6–9 years old), whereas it only had an effect on three parenting-related factors in older children (10–12 years old). It has been stated that parents play a major role in the development of healthy behavior of their primary schoolchildren [3, 12–14]. However, parental control begins to fade as the child grows older, and older primary schoolchildren get more freedom and decision-making power of their parents [54]. Therefore, it might be important that parents adopt specific parenting practices when their children are still young, and before an obesogenic lifestyle is deeply rooted. Furthermore, the results show that the intervention effects on parental self-efficacy related to PA, screen-time and healthy diet were all in parents of younger children (6–9 years old), whereas the intervention was only effective in changing parenting practices related to the three behaviors in older children (10–12 years old). An explanation could be that parents of younger children might still be searching on how they have to raise their children, suggesting that the parenting videos might have more effect on their self-efficacy concerning the parenting practices they have to adopt. It could therefore be argued that the intervention could focus on parents of younger children, but because parents often have more than one child in primary school and because there were also videos that affected parents of older children, it is recommended to promote the videos in all parents of primary school children. In contrast to children’s age, gender did not moderate the effect of the ‘Movie Models’ intervention on parenting practices and parental self-efficacy. While gender seemed to be the most important moderator in interventions according to the review of Yildirim and colleagues [55] (interventions appeared to work better for girls than for boys), the current study shows that there was no difference in intervention effect between parents with a daughter or a son. This way, the online videos do not have to be differentiated between parents of boys and girls.

A final aspect that we would like to elaborate on is that for parental self-efficacy concerning having vegetables available (in the overall model) and for some subgroup analyses, the results showed inverse effects of the intervention (e.g., parents of the intervention group had a significant decrease in their self-efficacy after 4 months). A potential explanation is that parents could have been reflective on the answers they gave on the questionnaire. After watching the online videos in which parenting practices are showed, parents might realize that certain parenting practices are more difficult to implement than thought before. This may cause a decreased score in the parental self-efficacy after the intervention. This phenomenon has been described before in a paper reporting on the short term effects of the UP4FUN intervention. This family-involved school-based intervention aiming at reducing and breaking up sitting time at home and breaking up sitting time in school among 10–12 year olds in Europe also showed significant effects on attitude for computer/games console use in favor of the control group [56].

### Strengths and limitations

The strengths of the current study include the novel intervention which is based on formative research and the multiple follow-up measurement points. Furthermore, the program is theory driven, and is based on difficult everyday life situations experienced by parents. Finally, in the intervention group, each video was watched by 93.3–100% of parents which shows a great adherence. However, this study was also subject to some limitations. First, recruitment was difficult, and drop-out was higher than expected. Also in other studies, recruitment of parents has been challenging [57], or a relatively high number of participants withdrew from the study [19]. However, it should be stated that parents had to complete a bulky questionnaire three times. So it is possible that parents dropped out because of the questionnaire and not because they did not like the videos. Furthermore, only 84 of 207 participants were recruited via primary schools and therefore, clusters could not be accounted for in the analyses. However, because the number of children recruited via the same school was low, we consider the chance of social support through the school clustering as limited. Secondly, the self-report questionnaires may have led to social desirability bias. Thirdly, single item measures of the parenting practices were used which could increase measurement error. Next, the intervention reached parents who were predominantly of high SES and with children of healthy BMI. Also the criterion that only parents with internet access could be included in the study might pose a threat to the generalizability of the study findings. Only a limited number of parents of children who might be in greatest need, were reached by the intervention. Therefore, extra effort will be necessary to ensure that the intervention also reaches at-risk parents. Furthermore, because the combination of social desirability bias and the highly educated group of participating parents may have resulted in ceiling effects, the intervention could be a more promising parenting program for at-risk parents who have lower baseline values. Nevertheless, it is possible that low SES parents do not care to participate in social media campaigns. Therefore, different strategies should be performed to work with these at-risk parents. ‘For example, low SES parents could be personally contacted by local community workers or local health providers who could encourage those parents to watch the videos. Finally, it is possible that a five-point answering format is not sensitive enough to measure the effect of the intervention. When parents already perform the parenting practice regularly at baseline, they will choose the answer category ‘often’ in the questionnaire. After the intervention, the parent might perform the behavior somewhat more, but not enough to report the highest level of frequency [58].

## Conclusions (main conclusions + importance and relevance)

The ‘Movie Models’ intervention was effective in increasing some important parenting practices and parental self-efficacy related to PA, screen-time and healthy diet in children. Therefore, the current intervention study is an important first step in promoting effective parenting-related factors. However, it should be further explored if the intervention is able to increase children’s healthy diet and PA and to decrease children’s screen-time on the long term. Furthermore, future studies should investigate if ‘Movie Models’ is a promising parenting program for at-risk parents with low SES and with overweight/obese children. Because the online parenting videos could be easily, inexpensively and widely distributed to large numbers of parents, its public health effect might be maximized.

## Additional files

Additional file 1: Table S1. Formulations and descriptive statistics of the questionnaire items of the specific parenting-related factors. (DOCX 26 kb)

Additional file 2: Table S4. Dataset. (XLSX 260 kb)

Additional file 3: Table S2. Two-way interaction effects on children’s behavior, and two-way and three-way interaction effects on parenting practices and parental self-efficacy. (DOCX 100 kb)

Additional file 4: Table S3. Two-way interaction effects stratified by moderator. (DOCX 44 kb)

## Abbreviations

ANOVA: Analysis of variance
BMI: Body mass index
PA: Physical activity
SES: Socio-economic status
T0: Baseline
T1: One month post baseline
T2: Four months post baseline
