# Supplementary material for: The effect of an online video intervention ‘Movie Models’ on specific parenting practices and parental self-efficacy related to children’s physical activity, screen-time and healthy diet: a quasi experimental study
Source: BMC Public Health. 2017 Apr 27;17:366. doi: 10.1186/s12889-017-4264-1 (PMC5408449; doi:10.1186/s12889-017-4264-1)
Supplement: Supplementary file 1 — Formulations and descriptive statistics of the questionnaire items of the specific parenting-related factors. (DOCX 26 kb) [file 12889_2017_4264_MOESM1_ESM.docx]

**Additional file 1: Table S1. Formulations and descriptive statistics of the questionnaire items of the specific parenting-related factors**

| **Beha-vior** | **Factor** | **Question item** | **Response alternatives** |
| --- | --- | --- | --- |
| PA | Availability | Do you have following sports materials at home for your child?   1. Bike 2. Tennis or badminton racket 3. Ball (basketball/volleyball/football) 4. Sport shoes 5. Skip rope 6. Roller-skates 7. Skateboard | 1 = no  2 = yes |
|  | Modeling | I am physically active nearby my child | 1 = never  2 = rarely  3 = sometimes  4 = often  5 = always |
|  | Motivating | I try to motivate my child to be physically active |  |
|  | Reinforcing | I reinforce my child when he/she is physically active |  |
|  | Giving choice | I let my child choose between different kinds of physical activities he/she wants to do |  |
|  | Involving | 1. I am physically active together with my child 2. I involve my child in household chores (e.g. cooking, cleaning, washing the dishes,…) |  |
|  | SE Availability | It is difficult for me to have sports materials at home for my child. | 1 = completely disagree  2 = mostly disagree  3 = sometimes disagree/sometimes agree  4 = mostly agree  5 = completely agree |
|  | SE Modeling | It is difficult for me to be physically active nearby my child. |  |
|  | SE Motivating | It is difficult for me to motivate my child to be physically active. |  |
|  | SE Reinforcing | It is difficult for me to reinforce my child when he/she is physically active. |  |
|  | SE Giving choice | It is difficult for me to let my child choose between different kinds of physical activities he/she wants to do. |  |
|  | SE Involving | 1. It is difficult for me to be physically active together with my child. 2. It is difficult for me to involve my child in household chores (e.g. cooking, cleaning, washing the dishes,…). |  |
| Screen-time | Permission | 1. My child has to ask for permission to play videogames, computer games, PlayStation, Nintendo,…   My child has to ask for permission to watch TV. | 1 = disagree  2 = agree |
|  | Rules | 1. In our family, there are rules about the moments (when and how long) my child is allowed to play videogames, computer games, PlayStation, Nintendo,… 2. In our family, there are rules about the moments (when and how long) my child is allowed to watch TV. |  |
|  | Being consistent | 1. The rules about when and how long my child is allowed to play videogames, computer games, PlayStation, Nintendo,…, are followed up. 2. The rules about when and how long my child is allowed to watch TV are followed up. | 1 = never  2 = rarely  3 = sometimes  4 = often  5 = always |
|  | Giving an explanation | 1. I explain to my child why there are rules about when he/she is allowed to play videogames, computer games, PlayStation, Nintendo,… 2. I explain to my child why there are rules about when he/she is allowed to watch TV. |  |
|  | Monitoring | 1. I monitor the time my child plays videogames, computer games, PlayStation, Nintendo,… 2. I monitor the time my child watches TV. |  |
|  | Modeling | 1. I limit my own playing of videogames, computer games, PlayStation, Nintendo,… nearby my child. 2. I limit my own TV-time nearby my child. |  |
|  | Motivating | 1. I try to motivate my child to play less videogames, computer games, PlayStation, Nintendo,… 2. I try to motivate my child to watch less TV. |  |
|  | SE Permission | 1. It is difficult for me to let my child ask for permission to play videogames, computer games, PlayStation, Nintendo,… 2. It is difficult for me to let my child ask for permission to watch TV. | 1 = completely disagree  2 = mostly disagree  3 = sometimes disagree/sometimes agree  4 = mostly agree  5 = completely agree |
|  | SE Rules | 1. It is difficult for me to apply rules about the moments my child is allowed to play videogames, computer games, PlayStation, Nintendo,… 2. It is difficult for me to apply rules about the moments my child is allowed to watch TV. |  |
|  | SE Being consistent | 1. It is difficult for me to follow up the rules about the moments my child is allowed to play videogames, computer games, PlayStation, Nintendo,… 2. It is difficult for me to follow up the rules about the moments my child is allowed to watch TV. |  |
|  | SE Giving an explanation | 1. It is difficult for me to explain to my child why there are rules about the moments my child is allowed to play videogames, computer games, PlayStation, Nintendo,… 2. It is difficult for me to explain to my child why there are rules about the moments my child is allowed to watch TV. |  |
|  | SE Monitoring games | It is difficult for me to monitor the time my child plays videogames, computer games, PlayStation, Nintendo,… |  |
|  | SE Modeling | 1. It is difficult for me to limit my own playing of videogames, computer games, PlayStation, Nintendo,… nearby my child. 2. It is difficult for me to limit my own TV-time nearby my child. |  |
|  | SE Motivating | 1. It is difficult for me to motivate my child to play less videogames, computer games, PlayStation, Nintendo,… 2. It is difficult for me to motivate my child to watch less TV. |  |
| Fruit | Modeling | I eat fruit in front of my child. | 1 = never  2 = rarely  3 = sometimes  4 = often  5 = always |
|  | Motivating | I try to motivate my child to eat more fruit. |  |
|  | Reinforcing | I reinforce my child for eating fruit. |  |
|  | Choice | I let my child choose between different kinds of fruit he/she wants to eat. |  |
|  | Availability | I have fruit available at home. |  |
|  | Involving | I involve my child in selecting fruit to buy in the supermarket. |  |
|  | SE Modeling | I find it difficult to eat fruit in front of my child. | 1 = completely disagree  2 = mostly disagree  3 = sometimes disagree/sometimes agree  4 = mostly agree  5 = completely agree |
|  | SE Motivating | I find it difficult to motivate my child to eat more fruit. |  |
|  | SE Reinforcing | I find it difficult to reinforce my child when he/she eats fruit. |  |
|  | SE Choice | I find it difficult to let my child choose between different kinds of fruit. |  |
|  | SE Availability | I find it difficult to have fruit available at home. |  |
|  | SE Involving | I find it difficult to involve my child in selecting fruit to buy in the supermarket. |  |
| Vegetables | Modeling | I eat vegetables in front of my child. | 1 = never  2 = rarely  3 = sometimes  4 = often  5 = always |
|  | Permissive-ness (at meals and between meals) | 1. My child can choose him/herself how many vegetables he/she wants to eat at meals. 2. My child can choose him/herself how many vegetables he/she wants to eat between meals. 3. My child can choose him/herself when he/she wants to eat vegetables between meals. |  |
|  | Motivating | I try to motivate my child to eat more vegetables. |  |
|  | Reinforcing | I reinforce my child for eating vegetables. |  |
|  | Choice | I prepare different kinds of vegetables so my child can choose which vegetables he/she wants to eat. |  |
|  | Availability | I have vegetables available at home. |  |
|  | Involving | I involve my child in selecting vegetables to buy in the supermarket. |  |
|  | SE Modeling | I find it difficult to eat vegetables in front of my child. | 1 = completely disagree  2 = mostly disagree  3 = sometimes disagree/sometimes agree  4 = mostly agree  5 = completely agree |
|  | SE Permissive-ness (at meals and between meals) | I find it difficult to let…   1. my child choose him/herself how many vegetables he/she wants to eat at meals. 2. my child choose him/herself how many vegetables he/she wants to eat between meals. 3. my child choose him/herself when he/she wants to eat vegetables between meals. |  |
|  | SE Motivating | I find it difficult to motivate my child to eat more vegetables. |  |
|  | SE Reinforcing | I find it difficult to reinforce my child when he/she eats vegetables. |  |
|  | SE Choice | I find it difficult to prepare different kinds of vegetables so my child can choose which vegetables he/she wants to eat. |  |
|  | SE Availability | I find it difficult to have vegetables available at home. |  |
|  | SE Involving | I find it difficult to involve my child in selecting vegetables to buy in the supermarket. |  |
| Water | Modeling | I drink water in front of my child. | 1 = never  2 = rarely  3 = sometimes  4 = often  5 = always |
|  | Permissive-ness (when and how much) | 1. My child can choose him/herself when he/she wants to drink water. 2. My child can choose him/herself how much water he/she wants to drink . |  |
|  | Motivating | I try to motivate my child to drink more water. |  |
|  | Reinforcing | I reinforce my child for drinking water. |  |
|  | Choice | I let my child choose between different kinds of water (plain water, sparkling water, with or without ice cubes). |  |
|  | Availability | I have drinkable water available at home. |  |
|  | SE Modeling | I find it difficult to drink water in front of my child. | 1 = completely disagree  2 = mostly disagree  3 = sometimes disagree/sometimes agree  4 = mostly agree  5 = completely agree |
|  | SE Permissive-ness (when and how much) | I find it difficult to let…   1. my child choose him/herself when he/she wants to drink water. 2. my child choose him/herself how much water he/she wants to drink . |  |
|  | SE Motivating | I find it difficult to motivate my child to drink more water. |  |
|  | SE Reinforcing | I find it difficult to reinforce my child for drinking water. |  |
|  | SE Choice | I find it difficult to let my child choose between different kinds of water. |  |
| Soft drinks | Rules | In our family, there are rules about the moments my child is allowed to drink soft drinks. | 1 = no  2 = yes |
|  | Being consistent | The rules about when my child is allowed to drink soft drinks, are followed up. | 1 = never  2 = rarely  3 = sometimes  4 = often  5 = always |
|  | Giving an explanation | I explain to my child why there are rules about when he/she is allowed to drink soft drinks. |  |
|  | Modeling | I limit my own consumption of soft drinks in front of my child. |  |
|  | SE Rules | I find it difficult to have rules about the moments my child is allowed to drink soft drinks. | 1 = completely disagree  2 = mostly disagree  3 = sometimes disagree/sometimes agree  4 = mostly agree  5 = completely agree |
|  | SE Being consistent | I find it difficult to follow up my rules about when my child is allowed to drink soft drinks. |  |
|  | SE Giving an explanation | I find it difficult to explain to my child why there are rules about when he/she is allowed to drink soft drinks. |  |
|  | SE Modeling | I find it difficult to limit my own consumption of soft drinks in front of my child. |  |
| Snacks | Rules | In our family, there are rules about the moments my child is allowed to eat snacks. | 1 = no  2 = yes |
|  | Being consistent | The rules about when my child is allowed to eat snacks, are followed up. | 1 = never  2 = rarely  3 = sometimes  4 = often  5 = always |
|  | Giving an explanation | I explain to my child why there are rules about when he/she is allowed to eat snacks. |  |
|  | Availability | I have snacks available at home. |  |
|  | SE Rules | I find it difficult to have rules about the moments my child is allowed to eat snacks. | 1 = completely disagree  2 = mostly disagree  3 = sometimes disagree/sometimes agree  4 = mostly agree  5 = completely agree |
|  | SE Being consistent | I find it difficult to follow up my rules about when my child is allowed to eat snacks. |  |
|  | SE Giving an explanation | I find it difficult to explain to my child why there are rules about when he/she is allowed to eat snacks. |  |

PA = physical activity, SE = self-efficacy
