# Supplementary material for: The effect of an online video intervention ‘Movie Models’ on specific parenting practices and parental self-efficacy related to children’s physical activity, screen-time and healthy diet: a quasi experimental study
Source: BMC Public Health. 2017 Apr 27;17:366. doi: 10.1186/s12889-017-4264-1 (PMC5408449; doi:10.1186/s12889-017-4264-1)
Supplement: Supplementary file 3 — Two-way interaction effects on children’s behavior, and two-way and three-way interaction effects on parenting practices and parental self-efficacy. (DOCX 100 kb) [file 12889_2017_4264_MOESM3_ESM.docx]

**Additional file 3: Table S2. Two-way interaction effects on children’s behavior, and two-way and three-way interaction effects on parenting practices and parental self-efficacy.**

| **Children’s behavior from T0 – T1** | | | | | | | | | | | | | | | | | |
| --- | --- | --- | --- | --- | --- | --- | --- | --- | --- | --- | --- | --- | --- | --- | --- | --- | --- |
| **Multivariate** |  |  |  |  | **Time x Group** | |  | | | | | | | | | | |
|  |  |  |  |  | F | P |  |  |  |  |  |  |  |  |  |  |  |
|  |  |  |  |  | 0.15 | 0.993 |  |  |  |  |  |  |  |  |  |  |  |
| **Univariate** |  | **n** | **T0** | **T1** | **Time x Group** | |  | | | | | | | | | | |
|  |  |  | Mean (SD) | Mean (SD) | F | P |  |  |  |  |  |  |  |  |  |  |  |
| PA (min/day) | IG | 45 | 49.43 (3.50) | 56.26 (4.40) | 0.52 | 0.474 |  | | | | | | | | | | |
|  | CG | 63 | 49.07 (3.90) | 50.49 (3.72) |  |  |  |  |  |  |  |  |  |  |  |  |  |
| Screen-time  (min/day) | IG | 45 | 105.83 (7.97) | 115.75 (11.75) | 0.09 | 0.759 |  |  |  |  |  |  |  |  |  |  |  |
|  | CG | 63 | 114.62 (9.33) | 118.72 (9.53) |  |  |  |  |  |  |  |  |  |  |  |  |  |
| Fruit (portions/week) | IG | 45 | 6.31 (0.89) | 6.58 (0.76) | 0.02 | 0.885 |  |  |  |  |  |  |  |  |  |  |  |
|  | CG | 63 | 6.14 (0.72) | 6.60 (0.89) |  |  |  |  |  |  |  |  |  |  |  |  |  |
| Vegetables (portions/week) | IG | 45 | 6.82 (0.39) | 7.27 (0.18) | 0.21 | 0.648 |  |  |  |  |  |  |  |  |  |  |  |
|  | CG | 63 | 6.63 (0.27) | 7.46 (0.45) |  |  |  |  |  |  |  |  |  |  |  |  |  |
| Water (portions/week) | IG | 45 | 11.73 (0.25) | 11.65 (0.42) | 0.07 | 0.799 |  |  |  |  |  |  |  |  |  |  |  |
|  | CG | 63 | 11.75 (0.33) | 11.93 (0.25) |  |  |  |  |  |  |  |  |  |  |  |  |  |
| Soft drinks (portions/week) | IG | 45 | 0.84 (0.45) | 0.83 (0.52) | 0.21 | 0.648 |  |  |  |  |  |  |  |  |  |  |  |
|  | CG | 63 | 1.05 (0.50) | 0.88 (0.29) |  |  |  |  |  |  |  |  |  |  |  |  |  |
| Snacks (portions/week) | IG | 45 | 1.75 (0.13) | 1.59 (0.14) | 0.27 | 0.607 |  |  |  |  |  |  |  |  |  |  |  |
|  | CG | 63 | 1.71 (0.12) | 1.68 (0.15) |  |  |  |  |  |  |  |  |  |  |  |  |  |
| **Children’s behavior from T0 – T2** | | | | | | | | | | | | | | | | | |
| **Multivariate** |  |  |  |  | **Time x Group** | |  | | | | | | | | | | |
|  |  |  |  |  | F | P |  |  |  |  |  |  |  |  |  |  |  |
|  |  |  |  |  | 0.79 | 0.594 |  |  |  |  |  |  |  |  |  |  |  |
| **Univariate** |  | **n** | **T0** | **T2** | **Time x Group** | |  | | | | | | | | | | |
|  |  |  | Mean (SD) | Mean (SD) | F | P |  |  |  |  |  |  |  |  |  |  |  |
| PA (min/day) | IG | 49 | 49.47 (3.60) | 56.07 (2.31) | 0.01 | 0.910 |  | | | | | | | | | | |
|  | CG | 63 | 51.73 (4.34) | 57.84 (3.73) |  |  |  |  |  |  |  |  |  |  |  |  |  |
| Screen-time  (min/day) | IG | 49 | 105.28 (9.49) | 104.27 (10.70) | 1.13 | 0.291 |  |  |  |  |  |  |  |  |  |  |  |
|  | CG | 63 | 129.06 (10.35) | 118.18 (11.94) |  |  |  |  |  |  |  |  |  |  |  |  |  |
| Fruit (portions/week) | IG | 49 | 5.97 (0.91) | 6.58 (0.88) | 0.22 | 0.642 |  |  |  |  |  |  |  |  |  |  |  |
|  | CG | 63 | 6.37 (0.82) | 7.27 (0.68) |  |  |  |  |  |  |  |  |  |  |  |  |  |
| Vegetables (portions/week) | IG | 49 | 6.46 (0.34) | 7.32 (0.39) | 2.17 | 0.144 |  |  |  |  |  |  |  |  |  |  |  |
|  | CG | 63 | 7.20 (0.35) | 7.39 (0.29) |  |  |  |  |  |  |  |  |  |  |  |  |  |
| Water (portions/week) | IG | 49 | 11.30 (0.28) | 11.95 (0.27) | 1.34 | 0.250 |  |  |  |  |  |  |  |  |  |  |  |
|  | CG | 63 | 11.79 (0.41) | 11.70 (0.37) |  |  |  |  |  |  |  |  |  |  |  |  |  |
| Soft drinks (portions/week) | IG | 49 | 0.91 (0.60) | 1.02 (0.73) | 2.02 | 0.158 |  |  |  |  |  |  |  |  |  |  |  |
|  | CG | 63 | 0.89 (0.46) | 0.81 (0.40) |  |  |  |  |  |  |  |  |  |  |  |  |  |
| Snacks (portions/week) | IG | 49 | 1.71 (0.10) | 1.69 (0.15) | 0.02 | 0.899 |  |  |  |  |  |  |  |  |  |  |  |
|  | CG | 63 | 1.60 (0.11) | 1.56 (0.12) |  |  |  |  |  |  |  |  |  |  |  |  |  |
| **Parenting practices from T0 – T1** | | | | | | | | | | | | | | | | | |
| **Univariate** |  | **n** | **T0** | **T1** | **Time x Group** | | **Time x Group x** **Age** | | | **Time x Group x Gender** | | | | **Time x Group x Parental SES** | | | |
|  |  |  | Mean (SD) | Mean (SD) | F | P | F | P | | F | | P | | F | | P | |
| Availability of sports material | IG | 55 | 5.51 (1.50) | 5.00 (2.38) | 0.04 | 0.845 | 0.00 | 0.987 | | 0.58 | | 0.449 | | 0.39 | | 0.536 | |
|  | CG | 80 | 5.85 (1.30) | 5.41 (2.15) |  |  |  |  | |  | |  | |  | |  | |
| Modeling of PA | IG | 45 | 3.40 (1.12) | 3.36 (1.09) | 0.08 | 0.781 | 0.82 | 0.368 | | 0.02 | | 0.903 | | 0.89 | | 0.348 | |
|  | CG | 69 | 3.23 (1.14) | 3.23 (1.18) |  |  |  |  | |  | |  | |  | |  | |
| Motivating concerning PA | IG | 43 | 3.91 (1.15) | 4.02 (0.83) | 0.09 | 0.763 | 0.51 | 0.477 | | 3.03 | | 0.085 | | 0.08 | | 0.781 | |
|  | CG | 69 | 3.67 (0.85) | 3.72 (0.98) |  |  |  |  | |  | |  | |  | |  | |
| Reinforcing concerning PA | IG | 44 | 3.70 (1.19) | 3.86 (0.98) | 0.82 | 0.368 | 0.55 | 0.460 | | 0.24 | | 0.627 | | 0.41 | | 0.522 | |
|  | CG | 70 | 3.50 (0.99) | 3.50 (0.83) |  |  |  |  | |  | |  | |  | |  | |
| Giving choice concerning PA | IG | 44 | 4.09 (0.68) | 4.11 (0.62) | 0.00 | 0.965 | 1.18 | 0.279 | | 0.56 | | 0.455 | | 0.77 | | 0.383 | |
|  | CG | 69 | 4.17 (0.80) | 4.20 (0.68) |  |  |  |  | |  | |  | |  | |  | |
| Involving in PA | IG | 44 | 3.05 (0.99) | 3.20 (0.77) | 0.01 | 0.934 | 0.08 | 0.784 | | 0.39 | | 0.535 | | 0.90 | | 0.345 | |
|  | CG | 70 | 2.43 (0.83) | 2.60 (0.84) |  |  |  |  | |  | |  | |  | |  | |
| Involving in household chores | IG | 44 | 3.20 (0.77) | 3.30 (0.77) | 4.78 | ***0.031*** | 3.09 | 0.082 | | 0.34 | | 0.559 | | 0.20 | | 0.653 | |
|  | CG | 70 | 3.04 (0.84) | 2.87 (0.90) |  |  |  |  | |  | |  | |  | |  | |
| Permission concerning TV | IG | 44 | 0.89 (0.32) | 0.89 (0.32) | 1.07 | 0.303 | 1.42 | 0.236 | | 0.17 | | 0.678 | | 0.05 | | 0.821 | |
|  | CG | 66 | 0.80 (0.40) | 0.86 (0.35) |  |  |  |  | |  | |  | |  | |  | |
| Permission concerning gaming | IG | 36 | 0.92 (0.28) | 0.89 (0.32) | 1.00 | 0.925 | 0.57 | 0.452 | | 1.94 | | 0.167 | | 0.58 | | 0.447 | |
|  | CG | 62 | 0.94 (0.25) | 0.90 (0.30) |  |  |  |  | |  | |  | |  | |  | |
| Rules concerning TV | IG | 41 | 0.78 (0.42) | 0.85 (0.36) | 0.00 | 0.971 | 2.02 | 0.159 | | 0.49 | | 0.486 | | 6.48 | | ***0.012*** | |
|  | CG | 66 | 0.80 (0.40) | 0.88 (0.33) |  |  |  |  | |  | |  | |  | |  | |
| Rules concerning gaming | IG | 37 | 0.78 (0.42) | 0.86 (0.35) | 1.16 | 0.285 | 2.88 | 0.093 | | 0.00 | | 0.968 | | 4.62 | | ***0.034*** | |
|  | CG | 61 | 0.75 (0.43) | 0.92 (0.28) |  |  |  |  | |  | |  | |  | |  | |
| Being consistent concerning TV | IG | 30 | 4.07 (0.58) | 4.07 (0.52) | 0.09 | 0.767 | 2.39 | 0.126 | | 0.17 | | 0.679 | | 0.18 | | 0.671 | |
|  | CG | 51 | 3.90 (0.50) | 3.94 (0.54) |  |  |  |  | |  | |  | |  | |  | |
| Being consistent concerning games | IG | 28 | 4.04 (0.79) | 4.11 (0.57) | 0.26 | 0.610 | 0.03 | 0.856 | | 1.07 | | 0.305 | | 2.96 | | 0.090 | |
|  | CG | 45 | 3.78 (0.88) | 3.76 (0.80) |  |  |  |  | |  | |  | |  | |  | |
| Giving an explanation concerning TV | IG | 30 | 4.20 (0.89) | 4.17 (0.75) | 0.00 | 0.969 | 3.05 | 0.085 | | 0.88 | | 0.350 | | 0.36 | | 0.552 | |
|  | CG | 50 | 4.06 (0.89) | 4.02 (0.71) |  |  |  |  | |  | |  | |  | |  | |
| Giving an explanation concerning gaming | IG | 28 | 4.29 (0.81) | 4.18 (0.82) | 0.29 | 0.593 | 0.59 | 0.445 | | 2.83 | | 0.097 | | 0.99 | | 0.324 | |
|  | CG | 45 | 4.07 (0.86) | 4.07 (0.69) |  |  |  |  | |  | |  | |  | |  | |
| Monitoring gaming | IG | 36 | 3.50 (1.30) | 3.86 (1.02) | 3.77 | 0.055 | 2.19 | 0.142 | | 0.11 | | 0.745 | | 3.42 | | 0.068 | |
|  | CG | 64 | 3.70 (1.06) | 3.67 (0.82) |  |  |  |  | |  | |  | |  | |  | |
| Modeling concerning TV | IG | 45 | 4.00 (0.95) | 3.91 (0.97) | 1.79 | 0.183 | 3.24 | 0.075 | | 1.18 | | 0.280 | | 5.50 | | ***0.021*** | |
|  | CG | 69 | 3.43 (1.16) | 3.59 (1.05) |  |  |  |  | |  | |  | |  | |  | |
| Modeling concerning gaming | IG | 45 | 3.89 (0.93) | 3.60 (0.96) | 2.42 | 0.123 | 0.01 | 0.905 | | 0.70 | | 0.403 | | 7.80 | | **0.006** | |
|  | CG | 69 | 3.35 (0.98) | 3.35 (0.94) |  |  |  |  | |  | |  | |  | |  | |
| Motivating concerning TV | IG | 43 | 3.65 (0.87) | 3.56 (0.91) | 3.61 | 0.060 | 1.63 | 0.205 | | 0.07 | | 0.793 | | 0.06 | | 0.815 | |
|  | CG | 69 | 3.20 (1.04) | 3.46 (0.92) |  |  |  |  | |  | |  | |  | |  | |
| Motivating concerning games | IG | 37 | 3.59 (1.04) | 3.51 (1.04) | 2.58 | 0.112 | 2.78 | 0.099 | | 0.12 | | 0.729 | | 0.03 | | 0.874 | |
|  | CG | 64 | 3.41 (0.94) | 3.66 (0.82) |  |  |  |  | |  | |  | |  | |  | |
| Modeling concerning fruit | IG | 48 | 4.69 (0.75) | 4.69 (0.51) | 0.41 | 0.523 | 0.50 | 0.482 | | 1.35 | | 0.249 | | 1.28 | | 0.261 | |
|  | CG | 70 | 4.56 (0.65) | 4.67 (0.74) |  |  |  |  | |  | |  | |  | |  | |
| Motivating concerning fruit | IG | 48 | 3.90 (1.26) | 4.21 (0.97) | 0.01 | 0.932 | 0.18 | 0.677 | | 0.00 | | 0.993 | | 0.0 | | 0.920 | |
|  | CG | 70 | 3.91 (1.16) | 4.20 (1.16) |  |  |  |  | |  | |  | |  | |  | |
| Reinforcing concerning fruit | IG | 47 | 3.74 (0.99) | 3.74 (0.97) | 0.87 | 0.353 | 0.07 | 0.800 | | 0.00 | | 0.951 | | 0.76 | | 0.385 | |
|  | CG | 67 | 3.42 (1.10) | 3.67 (0.91) |  |  |  |  | |  | |  | |  | |  | |
| Choice concerning fruit | IG | 49 | 3.76 (0.86) | 4.04 (0.71) | 2.45 | 0.121 | 0.03 | 0.870 | | 0.66 | | 0.418 | | 0.22 | | 0.638 | |
|  | CG | 70 | 4.04 (0.75) | 4.00 (0.76) |  |  |  |  | |  | |  | |  | |  | |
| Availability of fruit | IG | 47 | 4.77 (0.48) | 4.77 (0.43) | 0.20 | 0.659 | 0.02 | 0.902 | | 0.21 | | 0.648 | | 2.44 | | 0.121 | |
|  | CG | 69 | 4.93 (0.26) | 4.88 (0.32) |  |  |  |  | |  | |  | |  | |  | |
| Involving concerning fruit | IG | 48 | 3.42 (1.05) | 3.60 (1.03) | 1.66 | 0.201 | 0.82 | 0.369 | | 0.58 | | 0.447 | | 0.00 | | 0.980 | |
|  | CG | 70 | 3.44 (1.15) | 3.26 (1.16) |  |  |  |  | |  | |  | |  | |  | |
| Modeling concerning vegetables | IG | 47 | 4.87 (0.34) | 4.79 (0.41) | 2.01 | 0.159 | 0.03 | 0.860 | | 0.12 | | 0.733 | | 0.02 | | 0.898 | |
|  | CG | 71 | 4.82 (0.39) | 4.86 (0.35) |  |  |  |  | |  | |  | |  | |  | |
| Permissiveness concerning how much vegetables at meals | IG | 49 | 3.59 (1.21) | 3.53 (1.19) | 0.13 | 0.718 | 0.03 | 0.861 | | 2.40 | | 0.124 | | 0.18 | | 0.676 | |
|  | CG | 71 | 3.59 (1.23) | 3.65 (1.21) |  |  |  |  | |  | |  | |  | |  | |
| Permissiveness concerning when vegetables between meals | IG | 49 | 3.92 (1.12) | 4.29 (0.94) | 2.04 | 0.156 | 1.00 | 0.320 | | 0.15 | | 0.701 | | 0.03 | | 0.870 | |
|  | CG | 70 | 4.17 (0.95) | 4.17 (0.96) |  |  |  |  | |  | |  | |  | |  | |
| Permissiveness concerning how much vegetables between meals | IG | 48 | 3.81 (1.05) | 4.00 (0.99) | 0.63 | 0.428 | 2.19 | 0.142 | | 0.13 | | 0.720 | | 3.51 | | 0.063 | |
|  | CG | 70 | 4.07 (1.04) | 4.04 (1.06) |  |  |  |  | |  | |  | |  | |  | |
| Motivating concerning vegetables | IG | 49 | 4.02 (1.23) | 4.31 (0.87) | 0.03 | 0.872 | 1.28 | 0.261 | | 0.24 | | 0.625 | | 0.00 | | 0.993 | |
|  | CG | 70 | 3.94 (1.10) | 4.27 (0.96) |  |  |  |  | |  | |  | |  | |  | |
| Reinforcing concerning vegetables | IG | 48 | 3.81 (0.96) | 3.81 (0.98) | 0.17 | 0.685 | 1.04 | 0.310 | | 0.11 | | 0.747 | | 2.15 | | 0.146 | |
|  | CG | 69 | 3.65 (0.95) | 3.75 (0.81) |  |  |  |  | |  | |  | |  | |  | |
| Choice concerning vegetables | IG | 49 | 2.14 (0.96) | 2.55 (1.04) | 2.21 | 0.140 | 0.05 | 0.819 | | 1.38 | | 0.243 | | 0.53 | | 0.469 | |
|  | CG | 70 | 2.31 (0.99) | 2.33 (1.02) |  |  |  |  | |  | |  | |  | |  | |
| Availability of vegetables | IG | 48 | 4.81 (0.39) | 4.77 (0.43) | 0.06 | 0.801 | 1.91 | 0.170 | | 0.03 | | 0.856 | | 0.07 | | 0.799 | |
|  | CG | 70 | 4.84 (0.40) | 4.83 (0.38) |  |  |  |  | |  | |  | |  | |  | |
| Involving concerning vegetables | IG | 48 | 3.04 (1.15) | 3.29 (1.15) | 0.40 | 0.531 | 1.14 | 0.287 | | 0.67 | | 0.414 | | 1.16 | | 0.285 | |
|  | CG | 69 | 2.97 (1.38) | 3.01 (1.19) |  |  |  |  | |  | |  | |  | |  | |
| Modeling concerning water | IG | 49 | 4.76 (0.56) | 4.57 (0.76) | 2.72 | 0.102 | 0.04 | 0.839 | | 0.16 | | 0.691 | | 1.04 | | 0.311 | |
|  | CG | 71 | 4.49 (0.72) | 4.61 (0.62) |  |  |  |  | |  | |  | |  | |  | |
| Permissiveness concerning water (when) | IG | 50 | 4.72 (0.76) | 4.72 (0.61) | 0.12 | 0.735 | 0.36 | 0.550 | | 0.93 | | 0.337 | | 0.00 | | 0.982 | |
|  | CG | 70 | 4.81 (0.57) | 4.76 (0.69) |  |  |  |  | |  | |  | |  | |  | |
| Permissiveness concerning water (how much) | IG | 50 | 4.32 (0.98) | 4.58 (0.70) | 3.56 | 0.062 | 0.09 | 0.764 | | 1.44 | | 0.233 | | 4.32 | | ***0.040*** | |
|  | CG | 69 | 4.62 (0.71) | 4.48 (0.87) |  |  |  |  | |  | |  | |  | |  | |
| Motivating concerning water | IG | 49 | 4.20 (1.14) | 4.22 (0.99) | 3.01 | 0.086 | 1.07 | 0.304 | | 0.04 | | 0.851 | | 0.19 | | 0.663 | |
|  | CG | 70 | 3.74 (1.20) | 4.29 (1.16) |  |  |  |  | |  | |  | |  | |  | |
| Reinforcing concerning water | IG | 49 | 3.35 (1.23) | 3.41 (1.02) | 0.76 | 0.385 | 0.02 | 0.885 | | 1.25 | | 0.266 | | 0.03 | | 0.872 | |
|  | CG | 68 | 3.16 (1.19) | 3.49 (1.02) |  |  |  |  | |  | |  | |  | |  | |
| Choice concerning water | IG | 49 | 2.86 (1.49) | 3.33 (1.48) | 2.47 | 0.119 | 0.62 | 0.431 | | 0.00 | | 0.948 | | 0.04 | | 0.836 | |
|  | CG | 70 | 3.10 (1.50) | 2.97 (1.43) |  |  |  |  | |  | |  | |  | |  | |
| Availability of water | IG | 48 | 4.98 (0.14) | 4.94 (0.25) | 0.36 | 0.550 | 1.66 | 0.201 | | 3.03 | | 0.084 | | 0.37 | | 0.542 | |
|  | CG | 70 | 4.99 (0.12) | 4.97 (0.17) |  |  |  |  | |  | |  | |  | |  | |
| Rules concerning soft drinks | IG | 34 | 0.94 (0.24) | 0.97 (0.17) | 0.11 | 0.744 | 0.06 | 0.804 | | 0.64 | | 0.426 | | 1.45 | | 0.231 | |
|  | CG | 56 | 0.89 (0.31) | 0.89 (0.31) |  |  |  |  | |  | |  | |  | |  | |
| Being consistent concerning soft drinks | IG | 31 | 4.48 (0.51) | 4.45 (0.51) | 0.05 | 0.826 | 4.43 | ***0.039*** | | 1.57 | | 0.215 | | 0.56 | | 0.456 | |
|  | CG | 42 | 4.48 (0.51) | 4.40 (0.59) |  |  |  |  | |  | |  | |  | |  | |
| Giving an explanation concerning soft drinks | IG | 31 | 4.55 (0.77) | 4.26 (0.73) | 1.40 | 0.241 | 1.21 | 0.275 | | 0.14 | | 0.707 | | 0.73 | | 0.397 | |
|  | CG | 41 | 4.32 (0.96) | 4.34 (0.66) |  |  |  |  | |  | |  | |  | |  | |
| Modeling concerning soft drinks | IG | 47 | 4.28 (1.33) | 4.15 (1.50) | 1.20 | 0.277 | 0.84 | 0.362 | | 0.44 | | 0.507 | | 0.13 | | 0.718 | |
|  | CG | 70 | 4.26 (1.35) | 4.51 (1.02) |  |  |  |  | |  | |  | |  | |  | |
| Rules concerning snacks | IG | 51 | 0.92 (0.27) | 0.86 (0.35) | 0.61 | 0.437 | 0.00 | 0.974 | | 0.32 | | 0.572 | | 0.12 | | 0.728 | |
|  | CG | 73 | 0.92 (0.28) | 0.92 (0.28) |  |  |  |  | |  | |  | |  | |  | |
| Being consistent concerning snacks | IG | 38 | 4.21 (0.58) | 4.18 (0.56) | 1.05 | 0.309 | 1.05 | 0.309 | | 2.59 | | 0.111 | | 0.05 | | 0.825 | |
|  | CG | 50 | 4.10 (0.58) | 4.24 (0.56) |  |  |  |  | |  | |  | |  | |  | |
| Giving an explanation concerning snacks | IG | 38 | 4.45 (0.69) | 4.32 (0.66) | 0.11 | 0.746 | 0.03 | 0.863 | | 0.58 | | 0.447 | | 2.28 | | 0.135 | |
|  | CG | 51 | 4.27 (0.85) | 4.22 (0.67) |  |  |  |  | |  | |  | |  | |  | |
| Availability of snacks | IG | 48 | 4.08 (0.92) | 3.96 (0.92) | 0.31 | 0.582 | 1.76 | 0.187 | | 0.11 | | 0.745 | | 0.93 | | 0.337 | |
|  | CG | 70 | 4.04 (0.91) | 4.06 (1.03) |  |  |  |  | |  | |  | |  | |  | |
| **Parenting practices from T0 – T2** | | | | | | | | | | | | | | | | | |
| **Univariate** |  | **n** | **T0** | **T2** | **Time x Group** | | **Time x Group x Age** | | | | **Time x Group x Gender** | | | | **Time x Group x Parental SES** | | |
|  |  |  | Mean (SD) | Mean (SD) | F | P | F | | P | | F | | P | | F | | P |
| Availability of sports material | IG | 54 | 5.69 (1.30) | 5.69 (1.62) | 0.99 | 0.321 | 1.13 | | 0.290 | | 0.29 | | 0.594 | | 0.27 | | 0.603 |
|  | CG | 74 | 5.80 (1.37) | 5.43 (2.21) |  |  |  | |  | |  | |  | |  | |  |
| Modeling of PA | IG | 49 | 3.27 (1.13) | 3.27 (1.09) | 0.00 | 1.000 | 0.34 | | 0.563 | | 1.12 | | 0.291 | | 4.64 | | ***0.034*** |
|  | CG | 63 | 3.19 (1.19) | 3.19 (1.13) |  |  |  | |  | |  | |  | |  | |  |
| Motivating concerning PA | IG | 48 | 3.92 (1.05) | 4.00 (0.95) | 0.42 | 0.518 | 2.20 | | 0.141 | | 0.05 | | 0.825 | | 0.03 | | 0.859 |
|  | CG | 63 | 3.76 (0.80) | 3.73 (0.87) |  |  |  | |  | |  | |  | |  | |  |
| Reinforcing concerning PA | IG | 50 | 3.82 (1.06) | 3.90 (1.04) | 0.08 | 0.773 | 2.68 | | 0.105 | | 3.64 | | 0.059 | | 0.51 | | 0.478 |
|  | CG | 64 | 3.61 (0.88) | 3.64 (0.76) |  |  |  | |  | |  | |  | |  | |  |
| Giving choice concerning PA | IG | 50 | 4.10 (0.71) | 4.12 (0.59) | 0.00 | 0.979 | 0.93 | | 0.337 | | 1.37 | | 0.245 | | 0.34 | | 0.563 |
|  | CG | 62 | 4.13 (0.80) | 4.15 (0.60) |  |  |  | |  | |  | |  | |  | |  |
| Involving in PA | IG | 50 | 2.90 (1.07) | 3.18 (0.83) | 0.07 | 0.794 | 0.26 | | 0.613 | | 0.17 | | 0.680 | | 0.63 | | 0.429 |
|  | CG | 63 | 2.51 (0.80) | 2.75 (0.78) |  |  |  | |  | |  | |  | |  | |  |
| Involving in household chores | IG | 50 | 3.16 (0.77) | 3.26 (0.92) | 0.68 | 0.410 | 2.70 | | 0.103 | | 2.51 | | 0.116 | | 0.61 | | 0.437 |
|  | CG | 64 | 3.08 (0.90) | 3.06 (0.91) |  |  |  | |  | |  | |  | |  | |  |
| Permission TV | IG | 49 | 0.88 (0.33) | 0.86 (0.35) | 1.44 | 0.233 | 3.79 | | 0.054 | | 0.00 | | 0.972 | | 0.16 | | 0.690 |
|  | CG | 62 | 0.79 (0.41) | 0.84 (0.37) |  |  |  | |  | |  | |  | |  | |  |
| Permission gaming | IG | 41 | 0.90 (0.30) | 0.93 (0.26) | 3.58 | 0.061 | 1.52 | | 0.220 | | 0.62 | | 0.432 | | 0.75 | | 0.389 |
|  | CG | 56 | 0.93 (0.26) | 0.88 (0.33) |  |  |  | |  | |  | |  | |  | |  |
| Rules concerning TV | IG | 49 | 0.78 (0.42) | 0.88 (0.33) | 0.81 | 0.369 | 3.82 | | 0.053 | | 0.11 | | 0.743 | | 1.52 | | 0.221 |
|  | CG | 63 | 0.79 (0.41) | 0.83 (0.38) |  |  |  | |  | |  | |  | |  | |  |
| Rules concerning gaming | IG | 40 | 0.78 (0.42) | 0.78 (0.42) | 0.37 | 0.542 | 0.81 | | 0.370 | | 0.23 | | 0.631 | | 0.10 | | 0.756 |
|  | CG | 56 | 0.79 (0.41) | 0.84 (0.37) |  |  |  | |  | |  | |  | |  | |  |
| Being consistent concerning TV | IG | 37 | 4.03 (0.55) | 1.19 (0.66) | 0.73 | 0.396 | 6.75 | | ***0.011*** | | 1.06 | | 0.306 | | 0.72 | | 0.399 |
|  | CG | 45 | 3.89 (0.57) | 3.93 (0.58) |  |  |  | |  | |  | |  | |  | |  |
| Being consistent concerning gaming | IG | 28 | 4.18 (0.72) | 4.18 (0.61) | 0.00 | 1.000 | 2.93 | | 0.092 | | 1.82 | | 0.182 | | 0.91 | | 0.343 |
|  | CG | 39 | 3.79 (0.86) | 3.79 (0.73) |  |  |  | |  | |  | |  | |  | |  |
| Giving an explanation concerning TV | IG | 37 | 4.22 (0.85) | 4.24 (0.83) | 0.10 | 0.750 | 9.06 | | **0.004** | | 1.88 | | 0.175 | | 0.02 | | 0.891 |
|  | CG | 45 | 4.09 (0.85) | 4.18 (0.68) |  |  |  | |  | |  | |  | |  | |  |
| Giving an explanation concerning gaming | IG | 28 | 4.43 (0.69) | 4.32 (0.82) | 0.29 | 0.591 | 6.65 | | ***0.012*** | | 0.59 | | 0.444 | | 0.29 | | 0.593 |
|  | CG | 40 | 4.08 (0.86) | 4.08 (0.73) |  |  |  | |  | |  | |  | |  | |  |
| Monitoring gaming | IG | 40 | 3.40 (1.30) | 3.85 (0.92) | 1.84 | 0.178 | 2.63 | | 0.108 | | 2.40 | | 0.125 | | 5.10 | | ***0.026*** |
|  | CG | 54 | 3.57 (1.08) | 3.72 (1.00) |  |  |  | |  | |  | |  | |  | |  |
| Modeling concerning TV | IG | 50 | 3.96 (0.97) | 4.16 (0.98) | 1.57 | 0.213 | 0.31 | | 0.578 | | 0.01 | | 0.906 | | 5.14 | | ***0.025*** |
|  | CG | 63 | 3.49 (1.20) | 3.46 (1.15) |  |  |  | |  | |  | |  | |  | |  |
| Modeling concerning gaming | IG | 50 | 3.82 (0.96) | 3.68 (1.04) | 1.99 | 0.161 | 1.00 | | 0.321 | | 0.57 | | 0.451 | | 1.50 | | 0.224 |
|  | CG | 63 | 3.32 (0.98) | 3.44 (0.93) |  |  |  | |  | |  | |  | |  | |  |
| Motivating concerning TV | IG | 47 | 3.70 (0.86) | 3.89 (0.84) | 0.10 | 0.755 | 3.92 | | 0.050 | | 0.16 | | 0.688 | | 0.61 | | 0.438 |
|  | CG | 62 | 3.31 (0.97) | 3.55 (0.86) |  |  |  | |  | |  | |  | |  | |  |
| Motivating concerning gaming | IG | 41 | 3.61 (1.02) | 3.95 (0.89) | 1.50 | 0.224 | 0.08 | | 0.774 | | 0.00 | | 0.975 | | 0.51 | | 0.477 |
|  | CG | 54 | 3.44 (0.86) | 3.54 (0.88) |  |  |  | |  | |  | |  | |  | |  |
| Modeling concerning fruit | IG | 48 | 4.63 (0.79) | 4.71 (0.62) | 0.47 | 0.496 | 0.43 | | 0.515 | | 0.02 | | 0.892 | | 0.03 | | 0.860 |
|  | CG | 64 | 4.52 (0.78) | 4.69 (0.73) |  |  |  | |  | |  | |  | |  | |  |
| Motivating concerning fruit | IG | 51 | 4.04 (1.26) | 4.47 (1.03) | 8.00 | **0.006** | 0.24 | | 0.624 | | 0.18 | | 0.677 | | 1.69 | | 0.196 |
|  | CG | 66 | 4.08 (1.13) | 3.79 (1.21) |  |  |  | |  | |  | |  | |  | |  |
| Reinforcing concerning fruit | IG | 51 | 3.71 (0.97) | 3.63 (1.26) | 0.03 | 0.865 | 0.85 | | 0.358 | | 0.02 | | 0.895 | | 0.17 | | 0.685 |
|  | CG | 66 | 3.44 (1.05) | 3.39 (0.88) |  |  |  | |  | |  | |  | |  | |  |
| Choice concerning fruit | IG | 51 | 3.76 (0.86) | 3.76 (0.76) | 0.01 | 0.925 | 0.58 | | 0.449 | | 0.54 | | 0.463 | | 2.25 | | 0.136 |
|  | CG | 66 | 4.12 (0.78) | 4.14 (0.72) |  |  |  | |  | |  | |  | |  | |  |
| Availability of fruit | IG | 52 | 4.81 (0.45) | 4.87 (0.35) | 0.49 | 0.484 | 0.49 | | 0.486 | | 0.94 | | 0.335 | | 1.63 | | 0.204 |
|  | CG | 66 | 4.85 (0.40) | 4.85 (0.40) |  |  |  | |  | |  | |  | |  | |  |
| Involving concerning fruit | IG | 52 | 3.29 (1.07) | 3.56 (1.06) | 0.95 | 0.332 | 0.00 | | 0.977 | | 1.64 | | 0.202 | | 2.87 | | 0.093 |
|  | CG | 66 | 3.38 (1.13) | 3.45 (1.07) |  |  |  | |  | |  | |  | |  | |  |
| Modeling concerning vegetables | IG | 49 | 4.84 (0.37) | 4.80 (0.50) | 0.54 | 0.465 | 0.62 | | 0.434 | | 0.00 | | 0.981 | | 0.15 | | 0.699 |
|  | CG | 66 | 4.79 (0.45) | 4.82 (0.52) |  |  |  | |  | |  | |  | |  | |  |
| Permissiveness concerning how much vegetables at meals | IG | 50 | 3.60 (1.21) | 3.88 (0.96) | 0.00 | 0.986 | 0.07 | | 0.792 | | 1.43 | | 0.234 | | 0.29 | | 0.594 |
|  | CG | 67 | 3.33 (1.27) | 3.61 (1.27) |  |  |  | |  | |  | |  | |  | |  |
| Permissiveness concerning when vegetables between meals | IG | 49 | 3.78 (1.28) | 4.00 (1.08) | 0.94 | 0.335 | 0.30 | | 0.584 | | 0.31 | | 0.580 | | 1.10 | | 0.297 |
|  | CG | 67 | 4.12 (1.04) | 4.15 (1.00) |  |  |  | |  | |  | |  | |  | |  |
| Permissiveness concerning how much vegetables between meals | IG | 50 | 3.70 (1.22) | 3.94 (1.06) | 1.67 | 0.198 | 4.54 | | ***0.035*** | | 0.41 | | 0.524 | | 0.01 | | 0.930 |
|  | CG | 66 | 4.02 (1.10) | 4.00 (0.93) |  |  |  | |  | |  | |  | |  | |  |
| Motivating concerning vegetables | IG | 52 | 4.12 (1.11) | 4.37 (0.93) | 2.91 | 0.091 | 0.43 | | 0.515 | | 1.15 | | 0.286 | | 0.71 | | 0.401 |
|  | CG | 65 | 4.03 (1.08) | 3.92 (1.09) |  |  |  | |  | |  | |  | |  | |  |
| Reinforcing concerning vegetables | IG | 50 | 3.74 (0.94) | 3.68 (1.24) | 0.00 | 0.997 | 2.11 | | 0.149 | | 0.96 | | 0.329 | | 1.17 | | 0.282 |
|  | CG | 66 | 3.62 (0.97) | 3.56 (0.81) |  |  |  | |  | |  | |  | |  | |  |
| Choice concerning vegetables | IG | 52 | 2.15 (1.02) | 2.35 (0.99) | 0.03 | 0.869 | 1.11 | | 0.295 | | 0.02 | | 0.887 | | 1.99 | | 0.161 |
|  | CG | 65 | 2.46 (1.08) | 2.63 (1.13) |  |  |  | |  | |  | |  | |  | |  |
| Availability of vegetables | IG | 52 | 4.83 (0.38) | 4.79 (0.46) | 1.42 | 0.237 | 0.80 | | 0.374 | | 0.01 | | 0.945 | | 0.36 | | 0.547 |
|  | CG | 66 | 4.79 (0.45) | 4.83 (0.41) |  |  |  | |  | |  | |  | |  | |  |
| Involving concerning vegetables | IG | 52 | 3.02 (1.16) | 3.40 (1.19) | 1.84 | 0.178 | 1.03 | | 0.312 | | 0.02 | | 0.882 | | 1.85 | | 0.176 |
|  | CG | 66 | 3.00 (1.35) | 3.03 (1.12) |  |  |  | |  | |  | |  | |  | |  |
| Modeling concerning water | IG | 49 | 4.71 (0.58) | 4.65 (0.75) | 0.65 | 0.423 | 0.03 | | 0.869 | | 0.19 | | 0.667 | | 0.55 | | 0.459 |
|  | CG | 65 | 4.52 (0.71) | 4.57 (0.71) |  |  |  | |  | |  | |  | |  | |  |
| Permissiveness concerning water (when) | IG | 50 | 4.70 (0.79) | 4.58 (0.79) | 0.10 | 0.749 | 6.86 | | ***0.010*** | | 0.01 | | 0.914 | | 3.57 | | 0.061 |
|  | CG | 66 | 4.79 (0.60) | 4.71 (0.58) |  |  |  | |  | |  | |  | |  | |  |
| Permissiveness concerning water (how much) | IG | 51 | 4.37 (0.96) | 4.45 (0.86) | 0.55 | 0.460 | 2.56 | | 0.112 | | 0.02 | | 0.888 | | 0.18 | | 0.670 |
|  | CG | 66 | 4.58 (0.84) | 4.53 (0.66) |  |  |  | |  | |  | |  | |  | |  |
| Motivating concerning water | IG | 52 | 4.35 (1.12) | 4.44 (0.96) | 0.51 | 0.475 | 0.01 | | 0.941 | | 0.18 | | 0.670 | | 0.78 | | 0.380 |
|  | CG | 66 | 3.80 (1.28) | 4.05 (1.31) |  |  |  | |  | |  | |  | |  | |  |
| Reinforcing concerning water | IG | 52 | 3.37 (1.24) | 3.54 (1.20) | 0.75 | 0.390 | 0.50 | | 0.482 | | 1.50 | | 0.223 | | 0.56 | | 0.457 |
|  | CG | 65 | 3.09 (1.18) | 3.06 (1.03) |  |  |  | |  | |  | |  | |  | |  |
| Choice concerning water | IG | 52 | 2.87 (1.52) | 2.83 (1.41) | 0.56 | 0.455 | 0.02 | | 0.885 | | 0.13 | | 0.723 | | 6.08 | | ***0.015*** |
|  | CG | 66 | 3.24 (1.55) | 3.36 (1.41) |  |  |  | |  | |  | |  | |  | |  |
| Availability of water | IG | 51 | 4.96 (0.20) | 4.96 (0.20) | 0.00 | 1.000 | 0.00 | | 1.000 | | 0.00 | | 1.000 | | 3.12 | | 0.080 |
|  | CG | 66 | 4.98 (0.12) | 4.98 (0.12) |  |  |  | |  | |  | |  | |  | |  |
| Rules concerning soft drinks | IG | 42 | 0.88 (0.33) | 0.88 (0.33) | 0.11 | 0.742 | 0.09 | | 0.768 | | 1.29 | | 0.259 | | 0.69 | | 0.407 |
|  | CG | 54 | 0.89 (0.32) | 0.91 (0.29) |  |  |  | |  | |  | |  | |  | |  |
| Being consistent concerning soft drinks | IG | 34 | 4.38 (0.49) | 4.41 (0.61) | 0.00 | 0.950 | 0.09 | | 0.761 | | 1.75 | | 0.189 | | 8.43 | | **0.005** |
|  | CG | 47 | 4.45 (0.54) | 4.47 (0.58) |  |  |  | |  | |  | |  | |  | |  |
| Giving an explanation concerning soft drinks | IG | 34 | 4.59 (0.70) | 4.29 (0.72) | 0.17 | 0.677 | 4.08 | | ***0.047*** | | 0.60 | | 0.440 | | 0.88 | | 0.351 |
|  | CG | 46 | 4.59 (0.65) | 4.22 (0.92) |  |  |  | |  | |  | |  | |  | |  |
| Modeling concerning soft drinks | IG | 48 | 4.17 (1.43) | 4.54 (1.32) | 2.89 | 0.092 | 1.45 | | 0.231 | | 0.02 | | 0.884 | | 0.16 | | 0.694 |
|  | CG | 66 | 4.38 (1.31) | 4.36 (1.40) |  |  |  | |  | |  | |  | |  | |  |
| Rules concerning snacks | IG | 51 | 0.92 (0.27) | 0.92 (0.27) | 0.51 | 0.476 | 0.70 | | 0.405 | | 0.35 | | 0.556 | | 0.25 | | 0.615 |
|  | CG | 66 | 0.91 (0.29) | 0.94 (0.24) |  |  |  | |  | |  | |  | |  | |  |
| Being consistent concerning snacks | IG | 40 | 4.25 (0.59) | 4.20 (0.69) | 1.08 | 0.303 | 0.24 | | 0.629 | | 1.27 | | 0.262 | | 0.18 | | 0.670 |
|  | CG | 52 | 4.06 (0.57) | 4.15 (0.57) |  |  |  | |  | |  | |  | |  | |  |
| Giving an explanation concerning snacks | IG | 41 | 4.46 (0.71) | 4.07 (0.85) | 3.10 | 0.082 | 0.58 | | 0.450 | | 3.19 | | 0.077 | | 2.87 | | 0.094 |
|  | CG | 52 | 4.38 (0.69) | 4.29 (0.64) |  |  |  | |  | |  | |  | |  | |  |
| Availability of snacks | IG | 52 | 4.13 (0.91) | 3.88 (1.02) | 1.40 | 0.240 | 1.98 | | 0.162 | | 1.95 | | 0.165 | | 0.00 | | 0.993 |
|  | CG | 65 | 4.11 (0.89) | 4.02 (0.98) |  |  |  | |  | |  | |  | |  | |  |
| **Parental self-efficacy from T0 – T1** | | | | | | | | | | | | | | | | | |
| **Univariate** |  | **n** | **T0** | **T1** | **Time x Group** | | **Time x Group x Age** | | | | **Time x Group x Gender** | | | | **Time x Group x Parental SES** | | |
|  |  |  | Mean (SD) | Mean (SD) | F | P | F | | P | | F | | P | | F | | P |
| SE Availability of sports material | IG | 5 | 4.80 (0.45) | 5.00 (0.00) | 0.03 | 0.879 | 1.00 | | 0.363 | | 0.47 | | 0.526 | | . | | . |
|  | CG | 4 | 4.75 (0.50) | 5.00 (0.00) |  |  |  | |  | |  | |  | |  | |  |
| SE Modeling of PA | IG | 44 | 3.16 (1.38) | 3.64 (1.30) | 4.05 | ***0.046*** | 0.03 | | 0.862 | | 1.00 | | 0.319 | | 0.00 | | 0.990 |
|  | CG | 69 | 3.13 (1.40) | 3.25 (1.29) |  |  |  | |  | |  | |  | |  | |  |
| SE Motivating for PA | IG | 44 | 4.00 (1.10) | 4.09 (1.07) | 0.26 | 0.609 | 0.86 | | 0.356 | | 1.33 | | 0.251 | | 0.38 | | 0.537 |
|  | CG | 67 | 3.81 (1.10) | 3.99 (0.99) |  |  |  | |  | |  | |  | |  | |  |
| SE Reinforcing PA | IG | 44 | 4.39 (0.97) | 4.50 (0.82) | 0.29 | 0.592 | 0.09 | | 0.761 | | 0.48 | | 0.491 | | 2.31 | | 0.132 |
|  | CG | 67 | 4.18 (1.06) | 4.19 (0.96) |  |  |  | |  | |  | |  | |  | |  |
| SE Giving choice for PA | IG | 44 | 4.39 (0.75) | 4.25 (0.94) | 1.07 | 0.304 | 4.52 | | ***0.036*** | | 0.28 | | 0.598 | | 0.03 | | 0.875 |
|  | CG | 67 | 4.33 (0.88) | 4.36 (0.85) |  |  |  | |  | |  | |  | |  | |  |
| SE Involving in PA | IG | 44 | 3.70 (1.23) | 3.70 (1.15) | 2.68 | 0.104 | 0.90 | | 0.346 | | 1.63 | | 0.205 | | 0.62 | | 0.434 |
|  | CG | 66 | 2.91 (1.27) | 3.26 (1.24) |  |  |  | |  | |  | |  | |  | |  |
| SE Involving in household chores | IG | 44 | 3.80 (1.07) | 3.86 (1.05) | 0.82 | 0.368 | 0.70 | | 0.404 | | 0.16 | | 0.691 | | 0.05 | | 0.822 |
|  | CG | 67 | 3.46 (1.17) | 3.37 (1.23) |  |  |  | |  | |  | |  | |  | |  |
| SE Permission concerning TV | IG | 43 | 4.60 (0.82) | 4.49 (0.74) | 0.70 | 0.406 | 0.57 | | 0.450 | | 3.18 | | 0.077 | | 0.06 | | 0.813 |
|  | CG | 69 | 4.30 (1.02) | 4.33 (1.05) |  |  |  | |  | |  | |  | |  | |  |
| SE Permission concerning gaming | IG | 43 | 4.28 (1.01) | 4.42 (0.85) | 2.01 | 0.159 | 1.02 | | 0.315 | | 3.93 | | 0.050 | | 0.77 | | 0.381 |
|  | CG | 71 | 4.41 (0.85) | 4.34 (0.89) |  |  |  | |  | |  | |  | |  | |  |
| SE Rules concerning TV | IG | 39 | 4.08 (1.22) | 4.05 (1.17) | 0.17 | 0.683 | 0.00 | | 0.982 | | 0.12 | | 0.727 | | 0.35 | | 0.554 |
|  | CG | 71 | 4.06 (1.07) | 4.11 (1.02) |  |  |  | |  | |  | |  | |  | |  |
| SE Rules concerning gaming | IG | 43 | 3.93 (1.24) | 4.05 (1.13) | 0.73 | 0.395 | 0.03 | | 0.868 | | 0.43 | | 0.516 | | 0.24 | | 0.622 |
|  | CG | 71 | 4.13 (1.00) | 4.08 (0.95) |  |  |  | |  | |  | |  | |  | |  |
| SE Being consistent concerning TV | IG | 30 | 4.23 (0.86) | 4.30 (0.92) | 0.50 | 0.480 | 0.29 | | 0.589 | | 0.30 | | 0.587 | | 1.18 | | 0.282 |
|  | CG | 51 | 4.20 (0.89) | 4.14 (0.96) |  |  |  | |  | |  | |  | |  | |  |
| SE Being consistent concerning gaming | IG | 28 | 4.04 (1.10) | 4.29 (1.05) | 2.10 | 0.151 | 0.60 | | 0.442 | | 0.20 | | 0.653 | | 1.75 | | 0.191 |
|  | CG | 45 | 4.02 (1.12) | 3.93 (1.16) |  |  |  | |  | |  | |  | |  | |  |
| SE Giving an explanation concerning TV | IG | 29 | 4.38 (0.94) | 4.41 (0.82) | 0.95 | 0.333 | 1.67 | | 0.201 | | 0.00 | | 0.967 | | 0.33 | | 0.568 |
|  | CG | 51 | 4.43 (0.85) | 4.27 (0.85) |  |  |  | |  | |  | |  | |  | |  |
| SE Giving an explanation concerning gaming | IG | 27 | 4.30 (1.07) | 4.44 (0.75) | 1.12 | 0.293 | 0.00 | | 0.967 | | 0.16 | | 0.690 | | 0.78 | | 0.381 |
|  | CG | 45 | 4.47 (0.84) | 4.38 (0.78) |  |  |  | |  | |  | |  | |  | |  |
| SE Monitoring gaming | IG | 44 | 3.98 (1.34) | 4.18 (0.99) | 0.95 | 0.333 | 0.68 | | 0.411 | | 0.02 | | 0.901 | | 3.00 | | 0.086 |
|  | CG | 69 | 0.90 (1.13) | 3.93 (0.91) |  |  |  | |  | |  | |  | |  | |  |
| SE Modeling concerning TV | IG | 44 | 4.32 (0.86) | 4.23 (0.86) | 0.79 | 0.376 | 0.29 | | 0.593 | | 0.21 | | 0.652 | | 0.80 | | 0.373 |
|  | CG | 70 | 4.43 (0.84) | 4.19 (0.89) |  |  |  | |  | |  | |  | |  | |  |
| SE Modeling concerning gaming | IG | 44 | 4.07 (1.00) | 4.05 (1.01) | 1.11 | 0.294 | 6.25 | | ***0.014*** | | 0.00 | | 0.959 | | 1.05 | | 0.307 |
|  | CG | 70 | 4.16 (0.96) | 3.93 (0.98) |  |  |  | |  | |  | |  | |  | |  |
| SE Motivating concerning TV | IG | 42 | 3.98 (1.07) | 3.93 (1.09) | 0.00 | 0.994 | 0.02 | | 0.889 | | 2.37 | | 0.127 | | 0.19 | | 0.662 |
|  | CG | 65 | 3.85 (1.12) | 3.80 (1.03) |  |  |  | |  | |  | |  | |  | |  |
| SE Motivating concerning gaming | IG | 37 | 3.84 (1.21) | 3.92 (1.21) | 0.34 | 0.562 | 0.10 | | 0.755 | | 0.26 | | 0.610 | | 0.20 | | 0.657 |
|  | CG | 61 | 3.62 (1.19) | 3.59 (1.16) |  |  |  | |  | |  | |  | |  | |  |
| SE Modeling concerning fruit | IG | 47 | 4.74 (0.64) | 4.74 (0.61) | 0.01 | 0.923 | 0.24 | | 0.625 | | 1.54 | | 0.218 | | 0.56 | | 0.457 |
|  | CG | 68 | 4.72 (0.67) | 4.71 (0.62) |  |  |  | |  | |  | |  | |  | |  |
| SE Motivating concerning fruit | IG | 23 | 3.48 (1.27) | 3.57 (1.31) | 0.35 | 0.557 | 3.88 | | 0.054 | | 1.45 | | 0.234 | | 4.85 | | ***0.032*** |
|  | CG | 35 | 3.09 (1.44) | 3.46 (1.27) |  |  |  | |  | |  | |  | |  | |  |
| SE Reinforcing concerning fruit | IG | 46 | 4.30 (0.99) | 4.43 (0.81) | 0.46 | 0.498 | 0.36 | | 0.552 | | 0.12 | | 0.734 | | 1.27 | | 0.263 |
|  | CG | 67 | 4.24 (1.06) | 4.18 (1.00) |  |  |  | |  | |  | |  | |  | |  |
| SE Choice concerning fruit | IG | 46 | 4.20 (1.02) | 4.43 (0.83) | 1.12 | 0.292 | 10.06 | | **0.002** | | 0.11 | | 0.738 | | 0.98 | | 0.325 |
|  | CG | 67 | 4.46 (0.86) | 4.46 (0.64) |  |  |  | |  | |  | |  | |  | |  |
| SE Availability of fruit | IG | 47 | 4.70 (0.69) | 4.79 (0.55) | 0.79 | 0.375 | 5.64 | | ***0.019*** | | 0.00 | | 0.979 | | 3.85 | | 0.052 |
|  | CG | 67 | 4.84 (0.41) | 4.81 (0.47) |  |  |  | |  | |  | |  | |  | |  |
| SE Involving concerning fruit | IG | 47 | 4.17 (0.92) | 4.26 (0.99) | 0.59 | 0.446 | 0.29 | | 0.592 | | 0.60 | | 0.441 | | 0.28 | | 0.600 |
|  | CG | 68 | 4.41 (0.92) | 4.31 (0.92) |  |  |  | |  | |  | |  | |  | |  |
| SE Modeling concerning vegetables | IG | 46 | 4.87 (0.40) | 4.87 (0.40) | 0.87 | 0.353 | 0.28 | | 0.599 | | 5.69 | | ***0.019*** | | 0.20 | | 0.658 |
|  | CG | 68 | 4.88 (0.37) | 4.79 (0.48) |  |  |  | |  | |  | |  | |  | |  |
| SE Permissiveness concerning how much vegetables at meals | IG | 43 | 3.58 (1.31) | 3.65 (1.11) | 0.48 | 0.490 | 2.16 | | 0.145 | | 3.26 | | 0.074 | | 0.95 | | 0.331 |
|  | CG | 60 | 3.85 (1.10) | 4.15 (0.90) |  |  |  | |  | |  | |  | |  | |  |
| SE Permissiveness concerning when vegetables between meals | IG | 43 | 3.72 (1.32) | 4.33 (0.97) | 3.36 | 0.069 | 1.11 | | 0.296 | | 1.29 | | 0.259 | | 0.00 | | 0.998 |
|  | CG | 63 | 4.25 (0.97) | 4.30 (0.87) |  |  |  | |  | |  | |  | |  | |  |
| SE Permissiveness concerning how much vegetables between meals | IG | 42 | 3.74 (1.21) | 3.88 (1.09) | 0.10 | 0.751 | 1.32 | | 0.254 | | 0.02 | | 0.890 | | 1.04 | | 0.311 |
|  | CG | 59 | 4.02 (1.14) | 4.27 (0.94) |  |  |  | |  | |  | |  | |  | |  |
| SE Motivating concerning vegetables | IG | 35 | 3.23 (1.37) | 3.26 (1.29) | 0.24 | 0.628 | 0.54 | | 0.464 | | 0.44 | | 0.507 | | 0.00 | | 0.967 |
|  | CG | 51 | 3.29 (1.40) | 3.51 (1.17) |  |  |  | |  | |  | |  | |  | |  |
| SE Reinforcing concerning vegetables | IG | 45 | 4.29 (0.99) | 4.47 (0.79) | 0.46 | 0.497 | 0.33 | | 0.567 | | 0.59 | | 0.444 | | 3.26 | | 0.074 |
|  | CG | 68 | 4.26 (0.99) | 4.26 (0.92) |  |  |  | |  | |  | |  | |  | |  |
| SE Choice concerning vegetables | IG | 46 | 2.50 (1.41) | 2.80 (1.33) | 0.05 | 0.830 | 0.28 | | 0.595 | | 2.59 | | 0.110 | | 0.68 | | 0.411 |
|  | CG | 68 | 2.85 (1.49) | 3.07 (1.30) |  |  |  | |  | |  | |  | |  | |  |
| SE Availability of vegetables | IG | 47 | 4.72 (0.54) | 4.77 (0.56) | 0.01 | 0.932 | 9.63 | | **0.002** | | 0.21 | | 0.650 | | 1.26 | | 0.264 |
|  | CG | 67 | 4.73 (0.59) | 4.77 (0.56) |  |  |  | |  | |  | |  | |  | |  |
| SE Involving concerning vegetables | IG | 47 | 3.83 (1.11) | 4.13 (1.03) | 2.41 | 0.124 | 0.70 | | 0.406 | | 0.12 | | 0.734 | | 0.30 | | 0.584 |
|  | CG | 67 | 4.31 (0.97) | 4.21 (0.96) |  |  |  | |  | |  | |  | |  | |  |
| SE Modeling concerning water | IG | 47 | 4.74 (0.77) | 4.64 (0.82) | 0.01 | 0.921 | 0.63 | | 0.428 | | 0.50 | | 0.483 | | 0.17 | | 0.682 |
|  | CG | 68 | 4.78 (0.64) | 4.69 (0.63) |  |  |  | |  | |  | |  | |  | |  |
| SE Permissiveness concerning water (when) | IG | 44 | 4.11 (1.28) | 4.48 (0.93) | 2.56 | 0.113 | 0.02 | | 0.880 | | 0.00 | | 0.973 | | 0.19 | | 0.662 |
|  | CG | 63 | 4.62 (0.77) | 4.56 (0.74) |  |  |  | |  | |  | |  | |  | |  |
| SE Permissiveness concerning water (how much) | IG | 46 | 4.00 (1.28) | 4.24 (1.02) | 0.55 | 0.462 | 0.30 | | 0.587 | | 0.44 | | 0.509 | | 0.55 | | 0.461 |
|  | CG | 62 | 4.40 (0.95) | 4.42 (0.97) |  |  |  | |  | |  | |  | |  | |  |
| SE Motivating concerning water | IG | 45 | 3.76 (1.37) | 3.89 (1.34) | 0.86 | 0.355 | 0.10 | | 0.754 | | 1.67 | | 0.199 | | 0.16 | | 0.687 |
|  | CG | 62 | 3.82 (1.21) | 3.65 (1.23) |  |  |  | |  | |  | |  | |  | |  |
| SE Reinforcing concerning water | IG | 47 | 4.28 (1.12) | 4.34 (0.92) | 0.01 | 0.909 | 0.04 | | 0.850 | | 0.07 | | 0.794 | | 2.39 | | 0.125 |
|  | CG | 67 | 4.06 (1.15) | 4.09 (1.07) |  |  |  | |  | |  | |  | |  | |  |
| SE Choice concerning water | IG | 46 | 4.33 (1.30) | 4.41 (1.05) | 2.34 | 0.129 | 0.00 | | 0.967 | | 0.01 | | 0.926 | | 0.29 | | 0.592 |
|  | CG | 68 | 4.74 (0.68) | 4.43 (0.95) |  |  |  | |  | |  | |  | |  | |  |
| SE Rules concerning soft drinks | IG | 47 | 4.45 (0.93) | 4.57 (0.68) | 0.21 | 0.652 | 1.36 | | 0.246 | | 0.00 | | 0.967 | | 0.17 | | 0.679 |
|  | CG | 67 | 4.40 (0.91) | 4.42 (0.96) |  |  |  | |  | |  | |  | |  | |  |
| SE Being consistent concerning soft drinks | IG | 29 | 4.34 (0.77) | 4.48 (0.87) | 0.75 | 0.391 | 1.85 | | 0.179 | | 0.49 | | 0.486 | | 0.27 | | 0.608 |
|  | CG | 39 | 4.41 (0.72) | 4.31 (0.86) |  |  |  | |  | |  | |  | |  | |  |
| SE Giving an explanation concerning soft drinks | IG | 30 | 4.57 (0.63) | 4.63 (0.56) | 0.28 | 0.601 | 4.27 | | ***0.043*** | | 8.62 | | **0.005** | | 0.00 | | 0.968 |
|  | CG | 42 | 4.57 (0.74) | 4.50 (0.92) |  |  |  | |  | |  | |  | |  | |  |
| SE Modeling concerning soft drinks | IG | 47 | 4.11 (1.17) | 4.04 (1.12) | 0.00 | 0.983 | 0.06 | | 0.806 | | 0.03 | | 0.873 | | 0.03 | | 0.869 |
|  | CG | 68 | 4.26 (0.91) | 4.21 (0.87) |  |  |  | |  | |  | |  | |  | |  |
| SE Rules concerning snacks | IG | 49 | 4.47 (0.82) | 4.45 (0.91) | 0.09 | 0.761 | 0.31 | | 0.578 | | 0.07 | | 0.795 | | 1.00 | | 0.627 |
|  | CG | 54 | 4.39 (0.98) | 4.30 (0.90) |  |  |  | |  | |  | |  | |  | |  |
| SE Being consistent concerning snacks | IG | 36 | 4.19 (0.67) | 4.06 (1.09) | 0.30 | 0.583 | 0.72 | | 0.400 | | 0.12 | | 0.733 | | 0.03 | | 0.875 |
|  | CG | 48 | 4.23 (0.78) | 4.21 (0.80) |  |  |  | |  | |  | |  | |  | |  |
| SE Giving an explanation concerning snacks | IG | 36 | 4.47 (0.65) | 4.39 (0.99) | 1.01 | 0.318 | 1.21 | | 0.276 | | 1.09 | | 0.301 | | 0.00 | | 0.987 |
|  | CG | 42 | 4.36 (0.93) | 4.55 (0.74) |  |  |  | |  | |  | |  | |  | |  |
| **Parental self-efficacy from T0 – T2** | | | | | | | | | | | | | | | | | |
| **Univariate** |  | **n** | **T0** | **T2** | **Time x Group** | | **Time x Group x Age** | | | | **Time x Group x Gender** | | | | **Time x Group x Parental SES** | | |
|  |  |  | Mean (SD) | Mean (SD) | F | P | F | | P | | F | | P | | F | | P |
| SE Availability of sports material | IG | 12 | 4.75 (0.45) | 4.83 (0.39) | 0.16 | 0.698 | . | | . | | 0.01 | | 0.924 | | 0.94 | | 0.343 |
|  | CG | 13 | 4.85 (0.38) | 5.00 (0.00) |  |  |  | |  | |  | |  | |  | |  |
| SE Modeling of PA | IG | 50 | 3.18 (1.40) | 3.30 (1.20) | 0.05 | 0.823 | 0.10 | | 0.752 | | 0.06 | | 0.813 | | 3.68 | | 0.058 |
|  | CG | 66 | 3.14 (1.39) | 3.30 (1.32) |  |  |  | |  | |  | |  | |  | |  |
| SE Motivating for PA | IG | 50 | 4.00 (1.09) | 3.94 (1.06) | 1.58 | 0.211 | 0.38 | | 0.537 | | 0.12 | | 0.726 | | 0.06 | | 0.803 |
|  | CG | 65 | 3.95 (1.01) | 4.12 (0.88) |  |  |  | |  | |  | |  | |  | |  |
| SE Reinforcing PA | IG | 50 | 4.42 (0.93) | 4.36 (0.83) | 0.18 | 0.675 | 1.22 | | 0.273 | | 0.66 | | 0.418 | | 1.11 | | 0.295 |
|  | CG | 65 | 4.45 (0.90) | 4.32 (0.89) |  |  |  | |  | |  | |  | |  | |  |
| SE Giving choice for PA | IG | 50 | 4.38 (0.73) | 4.40 (0.73) | 0.00 | 0.947 | 0.00 | | 0.949 | | 0.44 | | 0.508 | | 0.64 | | 0.427 |
|  | CG | 64 | 4.41 (0.89) | 4.44 (0.69) |  |  |  | |  | |  | |  | |  | |  |
| SE Involving in PA | IG | 49 | 3.53 (1.32) | 3.67 (1.03) | 0.00 | 0.982 | 0.39 | | 0.534 | | 0.01 | | 0.937 | | 0.05 | | 0.832 |
|  | CG | 65 | 3.11 (1.32) | 3.25 (1.23) |  |  |  | |  | |  | |  | |  | |  |
| SE Involving in household chores | IG | 50 | 3.70 (1.11) | 3.84 (1.11) | 2.23 | 0.138 | 3.72 | | 0.056 | | 0.41 | | 0.522 | | 0.36 | | 0.552 |
|  | CG | 65 | 3.58 (1.17) | 3.46 (1.16) |  |  |  | |  | |  | |  | |  | |  |
| SE Permission concerning TV | IG | 48 | 4.58 (0.79) | 4.31 (1.06) | 0.48 | 0.488 | 1.93 | | 0.168 | | 0.96 | | 0.330 | | 0.00 | | 0.976 |
|  | CG | 64 | 4.39 (0.94) | 4.27 (1.01) |  |  |  | |  | |  | |  | |  | |  |
| SE Permission concerning gaming | IG | 50 | 4.30 (0.87) | 4.20 (1.09) | 0.78 | 0.380 | 4.25 | | ***0.042*** | | 1.06 | | 0.306 | | 0.44 | | 0.509 |
|  | CG | 65 | 4.35 (0.87) | 4.06 (1.03) |  |  |  | |  | |  | |  | |  | |  |
| SE Rules concerning TV | IG | 48 | 4.15 (1.15) | 3.90 (1.26) | 1.85 | 0.177 | 1.22 | | 0.271 | | 0.00 | | 0.953 | | 1.09 | | 0.298 |
|  | CG | 65 | 4.15 (0.99) | 4.18 (0.93) |  |  |  | |  | |  | |  | |  | |  |
| SE Rules concerning gaming | IG | 50 | 3.98 (1.19) | 3.80 (1.29) | 0.24 | 0.623 | 2.19 | | 0.142 | | 0.07 | | 0.798 | | 0.69 | | 0.408 |
|  | CG | 65 | 4.12 (0.96) | 4.05 (0.96) |  |  |  | |  | |  | |  | |  | |  |
| SE Being consistent concerning TV | IG | 36 | 4.19 (0.82) | 4.31 (0.89) | 0.45 | 0.505 | 0.81 | | 0.371 | | 1.12 | | 0.294 | | 1.29 | | 0.261 |
|  | CG | 43 | 4.23 (1.04) | 4.19 (0.93) |  |  |  | |  | |  | |  | |  | |  |
| SE Being consistent concerning gaming | IG | 27 | 4.22 (0.97) | 4.22 (1.01) | 0.23 | 0.636 | 1.00 | | 0.320 | | 0.11 | | 0.737 | | 2.55 | | 0.116 |
|  | CG | 37 | 4.24 (1.04) | 4.11 (0.99) |  |  |  | |  | |  | |  | |  | |  |
| SE Giving an explanation concerning TV | IG | 36 | 4.42 (0.84) | 4.42 (0.84) | 2.63 | 0.109 | 0.39 | | 0.537 | | 0.42 | | 0.517 | | 0.06 | | 0.811 |
|  | CG | 43 | 4.65 (0.61) | 4.37 (0.79) |  |  |  | |  | |  | |  | |  | |  |
| SE Giving an explanation concerning gaming | IG | 28 | 4.46 (0.92) | 4.36 (0.91) | 2.04 | 0.158 | 0.12 | | 0.732 | | 0.00 | | 0.954 | | 0.05 | | 0.824 |
|  | CG | 38 | 4.63 (0.71) | 4.24 (0.91) |  |  |  | |  | |  | |  | |  | |  |
| SE Monitoring gaming | IG | 48 | 3.94 (1.34) | 3.96 (1.15) | 0.02 | 0.878 | 1.81 | | 0.181 | | 0.49 | | 0.484 | | 2.13 | | 0.147 |
|  | CG | 64 | 3.91 (1.16) | 3.89 (1.04) |  |  |  | |  | |  | |  | |  | |  |
| SE Modeling concerning TV | IG | 50 | 4.36 (0.85) | 4.16 (1.04) | 0.01 | 0.946 | 1.56 | | 0.214 | | 0.12 | | 0.727 | | 0.02 | | 0.889 |
|  | CG | 66 | 4.38 (0.92) | 4.17 (1.00) |  |  |  | |  | |  | |  | |  | |  |
| SE Modeling concerning gaming | IG | 50 | 4.02 (1.00) | 4.08 (1.05) | 1.47 | 0.227 | 0.59 | | 0.442 | | 0.14 | | 0.713 | | 0.01 | | 0.912 |
|  | CG | 66 | 4.02 (0.98) | 3.82 (0.98) |  |  |  | |  | |  | |  | |  | |  |
| SE Motivating concerning TV | IG | 47 | 3.94 (1.11) | 3.87 (1.06) | 0.05 | 0.820 | 1.05 | | 0.308 | | 0.05 | | 0.818 | | 0.11 | | 0.744 |
|  | CG | 63 | 3.97 (1.00) | 3.95 (1.07) |  |  |  | |  | |  | |  | |  | |  |
| SE Motivating concerning gaming | IG | 42 | 3.83 (1.25) | 3.88 (1.11) | 0.42 | 0.517 | 0.69 | | 0.408 | | 1.23 | | 0.271 | | 1.12 | | 0.292 |
|  | CG | 55 | 3.82 (1.12) | 4.00 (1.00) |  |  |  | |  | |  | |  | |  | |  |
| SE Modeling concerning fruit | IG | 51 | 4.80 (0.57) | 4.80 (0.45) | 1.39 | 0.241 | 0.02 | | 0.884 | | 0.14 | | 0.708 | | 0.93 | | 0.336 |
|  | CG | 67 | 4.69 (0.76) | 4.52 (0.94) |  |  |  | |  | |  | |  | |  | |  |
| SE Motivating concerning fruit | IG | 32 | 3.25 (1.39) | 3.88 (1.29) | 1.44 | 0.233 | 0.96 | | 0.330 | | 0.49 | | 0.486 | | 3.79 | | 0.056 |
|  | CG | 43 | 3.30 (1.37) | 3.60 (1.22) |  |  |  | |  | |  | |  | |  | |  |
| SE Reinforcing concerning fruit | IG | 51 | 4.29 (1.01) | 4.27 (1.00) | 0.12 | 0.732 | 1.58 | | 0.211 | | 0.53 | | 0.468 | | 0.00 | | 0.983 |
|  | CG | 67 | 4.28 (0.97) | 4.33 (0.89) |  |  |  | |  | |  | |  | |  | |  |
| SE Choice concerning fruit | IG | 50 | 4.38 (0.97) | 4.44 (0.79) | 0.06 | 0.801 | 2.22 | | 0.139 | | 0.47 | | 0.496 | | 0.12 | | 0.729 |
|  | CG | 66 | 4.59 (0.78) | 4.61 (0.65) |  |  |  | |  | |  | |  | |  | |  |
| SE Availability of fruit | IG | 51 | 4.82 (0.52) | 4.76 (0.51) | 1.01 | 0.318 | 1.31 | | 0.256 | | 1.07 | | 0.303 | | 0.34 | | 0.564 |
|  | CG | 65 | 4.77 (0.49) | 4.82 (0.46) |  |  |  | |  | |  | |  | |  | |  |
| SE Involving concerning fruit | IG | 51 | 4.29 (0.88) | 4.45 (1.03) | 0.72 | 0.398 | 4.85 | | ***0.030*** | | 0.94 | | 0.334 | | 0.02 | | 0.899 |
|  | CG | 67 | 4.55 (0.82) | 4.54 (0.78) |  |  |  | |  | |  | |  | |  | |  |
| SE Modeling concerning vegetables | IG | 51 | 4.88 (0.38) | 4.84 (0.37) | 1.92 | 0.168 | 0.00 | | 0.997 | | 0.85 | | 0.358 | | 0.02 | | 0.895 |
|  | CG | 67 | 4.90 (0.39) | 4.67 (0.79) |  |  |  | |  | |  | |  | |  | |  |
| SE Permissiveness concerning how much vegetables at meals | IG | 47 | 3.62 (1.29) | 3.81 (1.21) | 1.68 | 0.198 | 1.04 | | 0.309 | | 0.39 | | 0.532 | | 0.35 | | 0.554 |
|  | CG | 57 | 3.77 (1.23) | 4.30 (0.96) |  |  |  | |  | |  | |  | |  | |  |
| SE Permissiveness concerning when vegetables between meals | IG | 48 | 3.92 (1.38) | 4.25 (1.04) | 1.43 | 0.235 | 2.57 | | 0.112 | | 0.54 | | 0.465 | | 0.44 | | 0.508 |
|  | CG | 61 | 4.46 (0.79) | 4.52 (0.74) |  |  |  | |  | |  | |  | |  | |  |
| SE Permissiveness concerning how much vegetables between meals | IG | 46 | 3.87 (1.19) | 4.04 (1.13) | 1.42 | 0.236 | 1.96 | | 0.165 | | 0.09 | | 0.767 | | 0.00 | | 0.963 |
|  | CG | 60 | 4.03 (1.15) | 4.48 (0.77) |  |  |  | |  | |  | |  | |  | |  |
| SE Motivating concerning vegetables | IG | 41 | 3.05 (1.38) | 3.66 (1.22) | 4.95 | ***0.029*** | 0.27 | | 0.608 | | 0.32 | | 0.576 | | 2.25 | | 0.137 |
|  | CG | 51 | 3.41 (1.37) | 3.51 (1.25) |  |  |  | |  | |  | |  | |  | |  |
| SE Reinforcing concerning vegetables | IG | 51 | 4.25 (1.02) | 4.31 (0.95) | 0.10 | 0.753 | 1.82 | | 0.179 | | 0.56 | | 0.455 | | 0.08 | | 0.775 |
|  | CG | 67 | 4.24 (1.05) | 4.36 (0.90) |  |  |  | |  | |  | |  | |  | |  |
| SE Choice concerning vegetables | IG | 51 | 2.73 (1.50) | 3.18 (1.24) | 1.42 | 0.235 | 0.35 | | 0.553 | | 0.67 | | 0.416 | | 1.25 | | 0.266 |
|  | CG | 66 | 3.12 (1.48) | 3.29 (1.43) |  |  |  | |  | |  | |  | |  | |  |
| SE Availability of vegetables | IG | 51 | 4.78 (0.46) | 4.69 (0.58) | 4.79 | ***0.031*** | 0.08 | | 0.785 | | 0.05 | | 0.823 | | 0.02 | | 0.903 |
|  | CG | 66 | 4.68 (0.64) | 4.79 (0.51) |  |  |  | |  | |  | |  | |  | |  |
| SE Involving concerning vegetables | IG | 51 | 4.08 (1.07) | 4.18 (1.18) | 1.30 | 0.257 | 6.00 | | ***0.016*** | | 1.91 | | 0.169 | | 2.20 | | 0.141 |
|  | CG | 66 | 4.48 (0.85) | 4.35 (0.95) |  |  |  | |  | |  | |  | |  | |  |
| SE Modeling concerning water | IG | 51 | 4.75 (0.80) | 4.78 (0.58) | 1.40 | 0.239 | 0.08 | | 0.776 | | 0.00 | | 0.965 | | 0.64 | | 0.424 |
|  | CG | 67 | 4.76 (0.74) | 4.60 (0.85) |  |  |  | |  | |  | |  | |  | |  |
| SE Permissiveness concerning water (when) | IG | 48 | 4.17 (1.33) | 4.56 (0.71) | 6.07 | ***0.015*** | 0.08 | | 0.777 | | 0.00 | | 0.996 | | 0.11 | | 0.742 |
|  | CG | 62 | 4.73 (0.58) | 4.63 (0.79) |  |  |  | |  | |  | |  | |  | |  |
| SE Permissiveness concerning water (how much) | IG | 48 | 4.04 (1.29) | 4.23 (1.13) | 0.58 | 0.448 | 0.57 | | 0.453 | | 1.86 | | 0.175 | | 2.29 | | 0.133 |
|  | CG | 64 | 4.45 (0.94) | 4.48 (0.89) |  |  |  | |  | |  | |  | |  | |  |
| SE Motivating concerning water | IG | 51 | 3.59 (1.43) | 3.88 (1.07) | 1.95 | 0.166 | 0.40 | | 0.530 | | 0.15 | | 0.699 | | 0.00 | | 0.969 |
|  | CG | 65 | 4.05 (1.12) | 4.02 (1.05) |  |  |  | |  | |  | |  | |  | |  |
| SE Reinforcing concerning water | IG | 51 | 4.25 (1.15) | 4.24 (1.07) | 0.14 | 0.713 | 2.35 | | 0.128 | | 0.06 | | 0.814 | | 0.32 | | 0.574 |
|  | CG | 66 | 4.18 (1.04) | 4.24 (1.04) |  |  |  | |  | |  | |  | |  | |  |
| SE Choice concerning water | IG | 51 | 4.35 (1.28) | 4.29 (1.10) | 0.76 | 0.385 | 0.06 | | 0.803 | | 0.27 | | 0.602 | | 0.31 | | 0.579 |
|  | CG | 67 | 4.84 (0.51) | 4.61 (0.82) |  |  |  | |  | |  | |  | |  | |  |
| SE Rules concerning soft drinks | IG | 51 | 4.35 (1.00) | 4.45 (0.94) | 1.79 | 0.183 | 0.36 | | 0.551 | | 0.95 | | 0.332 | | 2.01 | | 0.159 |
|  | CG | 66 | 4.45 (0.83) | 4.30 (1.01) |  |  |  | |  | |  | |  | |  | |  |
| SE Being consistent concerning soft drinks | IG | 34 | 4.53 (0.71) | 4.41 (0.74) | 1.18 | 0.280 | 0.01 | | 0.907 | | 0.35 | | 0.555 | | 0.01 | | 0.939 |
|  | CG | 43 | 4.51 (0.67) | 4.58 (0.66) |  |  |  | |  | |  | |  | |  | |  |
| SE Giving an explanation concerning soft drinks | IG | 33 | 4.64 (0.60) | 4.58 (0.56) | 0.30 | 0.583 | 0.35 | | 0.555 | | 1.05 | | 0.309 | | 0.70 | | 0.404 |
|  | CG | 46 | 4.78 (0.47) | 4.78 (0.42) |  |  |  | |  | |  | |  | |  | |  |
| SE Modeling concerning soft drinks | IG | 51 | 4.18 (1.13) | 4.35 (0.98) | 2.62 | 0.108 | 0.07 | | 0.794 | | 0.16 | | 0.694 | | 0.99 | | 0.322 |
|  | CG | 67 | 4.24 (1.07) | 4.09 (0.98) |  |  |  | |  | |  | |  | |  | |  |
| SE Rules concerning snacks | IG | 48 | 4.38 (0.84) | 4.17 (1.10) | 1.31 | 0.256 | 0.03 | | 0.860 | | 0.48 | | 0.488 | | 0.08 | | 0.784 |
|  | CG | 57 | 4.33 (0.97) | 4.35 (0.83) |  |  |  | |  | |  | |  | |  | |  |
| SE Being consistent concerning snacks | IG | 39 | 4.15 (0.71) | 4.26 (0.79) | 0.07 | 0.791 | 0.14 | | 0.710 | | 0.03 | | 0.857 | | 0.88 | | 0.351 |
|  | CG | 48 | 4.23 (0.81) | 4.27 (0.82) |  |  |  | |  | |  | |  | |  | |  |
| SE Giving an explanation concerning snacks | IG | 40 | 4.50 (0.72) | 4.58 (0.71) | 0.01 | 0.932 | 0.50 | | 0.481 | | 0.00 | | 0.978 | | 0.05 | | 0.821 |
|  | CG | 50 | 4.48 (0.84) | 4.54 (0.68) |  |  |  | |  | |  | |  | |  | |  |

IG=intervention group ; CG=control group; SE=self-efficacy

Significant p-values are indicated in bold; borderline significant p-values are indicated in bold italic
