# Supplementary material for: The effect of an online video intervention ‘Movie Models’ on specific parenting practices and parental self-efficacy related to children’s physical activity, screen-time and healthy diet: a quasi experimental study
Source: BMC Public Health. 2017 Apr 27;17:366. doi: 10.1186/s12889-017-4264-1 (PMC5408449; doi:10.1186/s12889-017-4264-1)
Supplement: Supplementary file 4 — Two-way interaction effects stratified by moderator. (DOCX 44 kb) [file 12889_2017_4264_MOESM4_ESM.docx]

**Additional file 4: Table S3. Two-way interaction effects stratified by moderator**

| **Age** | **Parenting practices from T0-T1** | | | | | |
| --- | --- | --- | --- | --- | --- | --- |
|  |  | **Time x Group** | |  | **T0** | **T1** |
|  |  | **F** | **P** |  | **Mean (SD)** | **Mean (SD)** |
| Being consistent concerning soft drink | 6-9 | 1.84 | 0.184 | I = 18 | 4.67 (0.49) | 4.44 (0.51) |
|  |  |  |  | C = 19 | 4.42 (0.51) | 4.53 (0.51) |
|  | 10-12 | 2.59 | 0.118 | I = 13 | 4.23 (0.44) | 4.46 (0.52) |
|  |  |  |  | C = 20 | 4.55 (0.51) | 4.35 (0.59) |
|  | **Parenting practices from T0-T2** | | | | | |
|  |  | **Time x Group** | |  | **T0** | **T2** |
|  |  | **F** | **P** |  | **Mean (SD)** | **Mean (SD)** |
| Being consistent concerning TV | 6-9 | 4.03 | 0.052 | I = 24 | 4.00 (0.51) | 4.29 (0.62) |
|  |  |  |  | C = 18 | 4.00 (0.77) | 3.83 (0.71) |
|  | 10-12 | 3.34 | 0.076 | I = 13 | 4.08 (0.64) | 4.00 (0.71) |
|  |  |  |  | C = 26 | 3.81 (0.40) | 4.00 (0.49) |
| Giving an explanation concerning TV | 6-9 | 3.58 | 0.066 | I = 24 | 4.17 (1.01) | 4.42 (0.72) |
|  |  |  |  | C = 18 | 4.22 (0.73) | 4.00 (0.69) |
|  | 10-12 | 5.37 | ***0.026*** | I = 13 | 4.31 (0.48) | 3.92 (0.95) |
|  |  |  |  | C = 26 | 4.00 (0.94) | 4.31 (0.68) |
| Giving an explanation concerning gaming | 6-9 | 2.01 | 0.167 | I = 16 | 4.50 (0.82) | 4.69 (0.48) |
|  |  |  |  | C = 14 | 4.07 (0.92) | 3.86 (0.66) |
|  | 10-12 | 5.04 | ***0.031*** | I = 12 | 4.33 (0.49) | 3.83 (0.94) |
|  |  |  |  | C = 25 | 4.08 (0.86) | 4.20 (0.76) |
| Giving an explanation concerning soft drink | 6-9 | 2.61 | 0.115 | I = 18 | 4.44 (0.86) | 4.50 (0.62) |
|  |  |  |  | C = 20 | 4.55 (0.76) | 4.20 (1.01) |
|  | 10-12 | 1.55 | 0.222 | I = 16 | 4.75 (0.45) | 4.06 (0.77) |
|  |  |  |  | C = 24 | 4.63 (0.58) | 4.25 (0.90) |
| Permissiveness concerning water (when) | 6-9 | 3.10 | 0.083 | I = 32 | 4.87 (0.34) | 4.56 (0.91) |
|  |  |  |  | C = 28 | 4.71 (0.81) | 4.75 (0.65) |
|  | 10-12 | 3.91 | 0.053 | I = 18 | 4.39 (1.20) | 4.61 (0.50) |
|  |  |  |  | C = 36 | 4.86 (0.35) | 4.69 (0.53) |
| Permissiveness concerning how much vegetables between meals | 6-9 | 0.15 | 0.704 | I = 33 | 3.82 (1.19) | 3.88 (1.05) |
|  |  |  |  | C = 27 | 3.63 (1.33) | 3.81 (1.04) |
|  | 10-12 | 11.70 | **0.001** | I = 17 | 3.47 (1.28) | 4.06 (1.09) |
|  |  |  |  | C = 37 | 4.32 (0.82) | 4.16 (0.83) |
|  | **Parental self-efficacy from T0-T1** | | | | | |
|  |  | **Time x Group** | |  | **T0** | **T1** |
|  |  | **F** | **P** |  | **Mean (SD)** | **Mean (SD)** |
| SE Modeling concerning gaming | 6-9 | 6.47 | ***0.014*** | I = 28 | 4.11 (1.03) | 4.32 (0.94) |
|  |  |  |  | C = 29 | 4.28 (1.00) | 3.86 (1.06) |
|  | 10-12 | 1.34 | 0.253 | I = 16 | 3.56 (0.96) | 4.00 (0.97) |
|  |  |  |  | C = 40 | 4.00 (0.93) | 4.08 (0.94) |
| SE Giving choice for PA | 6-9 | 3.75 | 0.058 | I = 28 | 4.50 (0.75) | 4.25 (0.93) |
|  |  |  |  | C = 27 | 4.19 (1.00) | 4.44 (0.89) |
|  | 10-12 | 1.02 | 0.317 | I = 16 | 4.19 (0.75) | 4.25 (1.00) |
|  |  |  |  | C = 40 | 4.43 (0.78) | 4.30 (0.82) |
| SE Giving an explanation concerning soft drinks | 6-9 | 1.32 | 0.259 | I = 17 | 4.65 (0.70) | 4.53 (0.62) |
|  |  |  |  | C = 19 | 4.53 (0.70) | 4.74 (0.56) |
|  | 10-12 | 2.91 | 0.098 | I = 13 | 4.46 (0.52) | 4.77 (0.44) |
|  |  |  |  | C = 20 | 4.70 (0.57) | 4.40 (0.88) |
| SE Choice concerning fruit | 6-9 | 8.38 | **0.005** | I = 29 | 4.00 (1.13) | 4.55 (0.74) |
|  |  |  |  | C = 31 | 4.74 (0.51) | 4.45 (0.62) |
|  | 10-12 | 2.89 | 0.096 | I = 17 | 4.53 (0.72) | 4.24 (0.97) |
|  |  |  |  | C = 33 | 4.21 (1.05) | 4.52 (0.67) |
| SE Availability of vegetables | 6-9 | 5.80 | ***0.019*** | I = 30 | 4.67 (0.61) | 4.90 (0.31) |
|  |  |  |  | C = 30 | 4.83 (0.46) | 4.63 (0.67) |
|  | 10-12 | 4.04 | 0.050 | I = 17 | 4.82 (0.39) | 4.53 (0.80) |
|  |  |  |  | C = 34 | 4.68 (0.64) | 4.85 (0.44) |
| SE Availability of fruit | 6-9 | 5.06 | ***0.028*** | I = 30 | 4.63 (0.81) | 4.87 (0.35) |
|  |  |  |  | C = 30 | 4.93 (0.25) | 4.77 (0.57) |
|  | 10-12 | 1.35 | 0.251 | I = 17 | 4.82 (0.39) | 4.65 (0.79) |
|  |  |  |  | C = 34 | 4.79 (0.41) | 4.82 (0.39) |
|  | **Parental self-efficacy from T0-T2** | | | | | |
|  |  | **Time x Group** | |  | **T0** | **T2** |
|  |  | **F** | **P** |  | **Mean (SD)** | **Mean (SD)** |
| SE Permission concerning gaming | 6-9 | 4.95 | ***0.030*** | I = 32 | 4.25 (0.98) | 4.41 (0.95) |
|  |  |  |  | C = 27 | 4.37 (0.88) | 3.93 (1.04) |
|  | 10-12 | 0.71 | 0.404 | I = 18 | 4.39 (0.92) | 3.83 (1.25) |
|  |  |  |  | C = 37 | 4.41 (0.80) | 4.14 (1.03) |
| SE Involving concerning vegetables | 6-9 | 5.56 | ***0.022*** | I = 32 | 3.91 (1.15) | 4.22 (1.10) |
|  |  |  |  | C = 28 | 4.68 (0.67) | 4.29 (1.08) |
|  | 10-12 | 1.23 | 0.273 | I = 19 | 4.37 (0.90) | 4.11 (1.33) |
|  |  |  |  | C = 37 | 4.35 (0.95) | 4.41 (0.86) |
| SE Involving concerning fruit | 6-9 | 3.99 | 0.050 | I = 32 | 4.19 (0.90) | 4.53 (0.84) |
|  |  |  |  | C = 28 | 4.71 (0.71) | 4.46 (1.04) |
|  | 10-12 | 1.25 | 0.268 | I = 19 | 4.47 (0.84) | 4.32 (1.29) |
|  |  |  |  | C = 38 | 4.45 (0.89) | 4.61 (0.55) |
| **Gender** | **Parental self-efficacy from T0-T1** | | | | | |
|  |  | **Time x Group** | |  | **T0** | **T1** |
|  |  | **F** | **P** |  | **Mean (SD)** | **Mean (SD)** |
| SE Giving an explanation concerning soft drinks | Boys | 4.24 | ***0.047*** | I = 18 | 4.67 (0.59) | 4.50 (0.62) |
|  |  |  |  | C = 18 | 4.44 (0.86) | 4.89 (0.32) |
|  | Girls | 4.44 | ***0.043*** | I = 12 | 4.42 (0.67) | 4.83 (0.39) |
|  |  |  |  | C = 24 | 4.67 (0.64) | 4.21 (1.10) |
| SE Modeling concerning vegetables | Boys | 6.38 | ***0.014*** | I = 28 | 4.82 (0.48) | 4.93 (0.38) |
|  |  |  |  | C = 34 | 4.94 (0.24) | 4.76 (0.50) |
|  | Girls | 1.13 | 0.293 | I = 18 | 4.94 (0.24) | 4.78 (0.43) |
|  |  |  |  | C = 34 | 4.82 (0.46) | 4.82 (0.46) |
| **Parental SES** | **Parenting practices from T0-T1** | | | | | |
|  |  | **Time x Group** | |  | **T0** | **T1** |
|  |  | **F** | **P** |  | **Mean (SD)** | **Mean (SD)** |
| Rules concerning TV | Low SES | 4.05 | 0.063 | I = 6 | 0.33 (0.52) | 0.83 (0.41) |
|  |  |  |  | C = 11 | 0.91 (0.30) | 1.00 (0.00) |
|  | High SES | 1.03 | 0.314 | I = 35 | 0.86 (0.36) | 0.86 (0.36) |
|  |  |  |  | C = 55 | 0.78 (0.42) | 0.85 (0.36) |
| Rules concerning gaming | Low SES | 2.18 | 0.162 | I = 5 | 0.40 (0.55) | 0.80 (0.45) |
|  |  |  |  | C = 11 | 0.82 (0.40) | 0.91 (0.30) |
|  | High SES | 3.34 | 0.071 | I = 32 | 0.84 (0.37) | 0.88 (0.34) |
|  |  |  |  | C = 50 | 0.74 (0.44) | 0.92 (0.27) |
| Modeling concerning TV | Low SES | 5.75 | ***0.028*** | I = 8 | 3.88 (1.25) | 3.50 (1.07) |
|  |  |  |  | C = 11 | 2.64 (0.67) | 3.45 (1.21) |
|  | High SES | 0.10 | 0.752 | I = 37 | 4.03 (0.90) | 4.00 (0.94) |
|  |  |  |  | C = 58 | 3.59 (1.17) | 3.62 (1.02) |
| Modeling concerning gaming | Low SES | 6.67 | ***0.019*** | I = 8 | 4.50 (0.76) | 3.38 (1.06) |
|  |  |  |  | C = 11 | 2.91 (0.54) | 3.18 (0.98) |
|  | High SES | 0.09 | 0.765 | I = 37 | 3.76 (0.93) | 3.65 (0.95) |
|  |  |  |  | C = 58 | 3.43 (1.03) | 3.38 (0.93) |
| Permissiveness concerning water (how much) | Low SES | 0.84 | 0.371 | I = 9 | 4.33 (0.12) | 4.33 (1.00) |
|  |  |  |  | C = 10 | 4.30 (1.06) | 4.90 (0.32) |
|  | High SES | 7.16 | **0.009** | I = 41 | 4.32 (0.96) | 4.63 (0.62) |
|  |  |  |  | C = 59 | 4.68 (0.63) | 4.41 (0.91) |
|  | **Parenting practices from T0-T2** | | | | | |
|  |  | **Time x Group** | |  | **T0** | **T2** |
|  |  | **F** | **P** |  | **Mean (SD)** | **Mean (SD)** |
| Modeling of PA | Low SES | 1.93 | 0.183 | I = 11 | 3.27 (1.27) | 2.64 (1.03) |
|  |  |  |  | C = 8 | 3.13 (1.25) | 3.13 (1.25) |
|  | High SES | 1.59 | 0.211 | I = 38 | 3.26 (1.11) | 3.45 (1.06) |
|  |  |  |  | C = 55 | 3.20 (1.19) | 3.20 (1.13) |
| Monitoring gaming | Low SES | 3.27 | 0.094 | I = 7 | 3.86 (1.46) | 3.43 (1.72) |
|  |  |  |  | C = 8 | 3.62 (0.92) | 4.00 (0.54) |
|  | High SES | 4.68 | ***0.034*** | I = 33 | 3.30 (1.26) | 3.94 (0.66) |
|  |  |  |  | C = 46 | 3.57 (1.11) | 3.67 (1.06) |
| Modeling concerning TV | Low SES | 3.46 | 0.079 | I = 12 | 3.75 (1.22) | 3.92 (1.24) |
|  |  |  |  | C = 8 | 2.75 (0.71) | 3.63 (0.74) |
|  | High SES | 3.34 | 0.071 | I = 38 | 4.03 (0.88) | 4.24 (0.88) |
|  |  |  |  | C = 55 | 3.60 (1.23) | 3.44 (1.20) |
| Being consistent concerning soft drinks | Low SES | 4.31 | 0.065 | I = 6 | 4.33 (0.52) | 3.83 (0.75) |
|  |  |  |  | C = 6 | 4.33 (0.52) | 4.67 (0.52) |
|  | High SES | 1.69 | 0.198 | I = 28 | 4.39 (0.50) | 4.54 (0.51) |
|  |  |  |  | C = 41 | 4.46 (0.55) | 4.44 (0.59) |
| Choice concerning water | Low SES | 5.87 | ***0.026*** | I = 12 | 3.50 (1.45) | 2.83 (1.59) |
|  |  |  |  | C = 8 | 3.75 (1.17) | 4.38 (0.52) |
|  | High SES | 0.18 | 0.670 | I = 40 | 2.68 (1.51) | 3.82 (1.38) |
|  |  |  |  | C = 58 | 3.17 (1.59) | 3.22 (1.44) |
|  | **Parental self-efficacy from T0-T1** | | | | | |
|  |  | **Time x Group** | |  | **T0** | **T1** |
|  |  | **F** | **P** |  | **Mean (SD)** | **Mean (SD)** |
| SE Motivating concerning fruit | Low SES | 1.88 | 0.220 | I = 5 | 2.80 (1.79) | 3.80 (1.30) |
|  |  |  |  | C = 3 | 4.67 (0.58) | 3.33 (2.08) |
|  | High SES | 2.03 | 0.160 | I = 18 | 3.67 (1.08) | 3.50 (1.34) |
|  |  |  |  | C = 32 | 2.94 (1.41) | 3.47 (1.22) |

IG=intervention group ; CG=control group; SE=self-efficacy

Significant p-values are indicated in bold; borderline significant p-values are indicated in bold italic
